# BioPhytMol: a drug discovery community resource on anti-mycobacterial phytomolecules and plant extracts

**DOI:** 10.1186/s13321-014-0046-2

**Published:** 2014-10-11

**Authors:** Arun Sharma, Prasun Dutta, Maneesh Sharma, Neeraj Kumar Rajput, Bhavna Dodiya, John J Georrge, Trupti Kholia, Anshu Bhardwaj, OSDD Consortium

**Affiliations:** 1 Open Source Drug Discovery (OSDD) Unit, Council of Scientific and Industrial Research, New Delhi, India; 2 Department of Applied Mathematics and Bioinformatics, Faculty of Technology and Engineering, The Maharaja Sayajirao University of Baroda, Vadodara, Gujarat India; 3 Department of Bioinformatics, Christ College, Rajkot, Gujarat India; 4 St. Stephens College, University of Delhi, New Delhi, India; 5 Department of Biochemistry and Molecular Biology, University Clinic of Bonn (UKB), University of Bonn, Bonn, Germany

**Keywords:** BioPhytMol, Anti-tubercular, TB, Tuberculosis, Anti-TB, Anti-mycobacterial, Drug discovery, Crowdsourcing

## Abstract

**Background:**

Tuberculosis (TB) is the second leading cause of death from a single infectious organism, demanding attention towards discovery of novel anti-tubercular compounds. Natural products or their derivatives have provided more than 50% of all existing drugs, offering a chemically diverse space for discovery of novel drugs.

**Description:**

BioPhytMol has been designed to systematically curate and analyze the anti-mycobacterial natural product chemical space. BioPhytMol is developed as a drug-discovery community resource with anti-mycobacterial phytomolecules and plant extracts. Currently, it holds 2582 entries including 188 plant families (692 genera and 808 species) from global flora, manually curated from literature. In total, there are 633 phytomolecules (with structures) curated against 25 target mycobacteria. Multiple analysis approaches have been used to prioritize the library for drug-like compounds, for both whole cell screening and target-based approaches. In order to represent the multidimensional data on chemical diversity, physiochemical properties and biological activity data of the compound library, novel approaches such as the use of circular graphs have been employed.

**Conclusion:**

BioPhytMol has been designed to systematically represent and search for anti-mycobacterial phytochemical information. Extensive compound analyses can also be performed through web-application for prioritizing drug-like compounds. The resource is freely available online at http://ab-openlab.csir.res.in/biophytmol/.

**Graphical Abstract:**
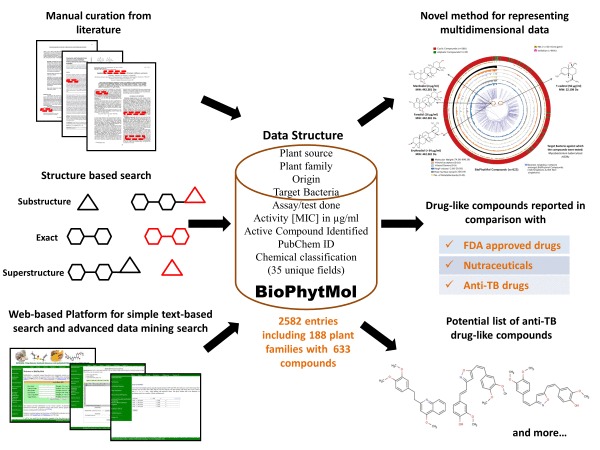
BioPhytMol: a drug discovery community resource on anti-mycobacterial phytomolecules and plant extracts generated using Crowdsourcing. The platform comprises of manually curated data on antimycobacterial natural products along with tools to perform structure similarity and visualization. The platform allows for prioritization of drug like natural products for antimycobacterial drug discovery.

**Electronic supplementary material:**

The online version of this article (doi:10.1186/s13321-014-0046-2) contains supplementary material, which is available to authorized users.

## Background

According to the WHO Global Tuberculosis report, 2013, TB alone was responsible for nearly 1.3 million deaths and 8.6 million new cases in 2012 [1]. The current TB treatment regimen is lengthy and multi-therapy based. Over time, TB causing bacteria i.e., *Mycobacterium tuberculosis* (Mtb) has acquired resistance against existing antibiotics leading to multi-drug resistant (MDR), extensively drug resistant (XDR) and totally drug-resistant (TDR) forms [2]. The long-duration therapies also possess various side-effects and toxicity issues [3]. Bedaquiline has been recently approved by United States Food and Drug Administration (FDA) in December 2012, almost after 50 years of the discovery of the last anti-tubercular (anti-TB) drug. This drug comes with a black box warning of induction of arrhythmia followed by the risk of developing resistance if used improperly [4]. There are other anti-TB drugs too in various stages of development [3]-[5]. However, there is a need to discover novel, highly effective, less-toxic anti-TB drugs having new mechanism of action. There is an increasing demand to explore new chemical space given the emergence of drug-resistance even for newly developed therapies. Exploring natural product (NP) space may prove to be a valuable source of new and less-toxic anti-TB drugs [5],[6]. Analysis of the NP chemical space has led to the identification of novel scaffolds [7] and increased chance of discovering compounds possessing novel mechanism of action [8]. The overall chemical space covered by synthetic compounds is limited as compared to natural compounds given their higher chemical diversity [9]. More than 50% of FDA approved drugs have been either NPs or NP derivatives [10]. For example, the recent FDA approved drug, Fulyzaq (http://www.fda.gov/NewsEvents/Newsroom/PressAnnouncements/ucm333701.htm), is the first anti-diarrheal drug for adult HIV/AIDS patients derived from the plant *Croton lechleri*. Also, of currently known 28 anti-TB drugs [2], eight drugs i.e., Rifampicin, Rifapentine, Streptomycin, Kanamycin, Amikacin, Capreomycin, Viomycin and Cycloserine have been derived from microbial sources (NPs) (see Additional file 1: Table S1). Many experimentally tested anti-mycobacterial NPs are scattered throughout scientific literature [11],[12]. In order to systematically evaluate their anti-mycobacterial properties, this data needs to be systematically collated and analyzed. A number of pathway and cheminformatics tools for TB drug discovery have been developed but are not exclusively for plant derived anti-mycobacterial compounds [13],[14]. At present, Universal Natural Products Database (UNPD) is the largest freely available resource for NPs and holds a total of 197201 NPs of plants, animals and microbial origin [15]. The various other NP databases and chemical libraries freely available for public use are NuBBE [16], CamMedNP [17], SuperNatural [18], HIT [19], NPACT [20], TCM Database@Taiwan [21], TCMID [22], HIM-herbal ingredients in-vivo metabolism database [23], AfroDB [24] and ConMedNP [25], to name a few. However, information related to phytomolecules and plant extracts against mycobacterial strains is very limited. Moreover, such data resources should be equipped with analysis tools to prioritize compounds for early stage drug discovery, which is missing. Furthermore, all the resources may follow a standardized global ontology so that the data shared by individuals or laboratories is interoperable and facilitate implementation of the semantic web features.

BioPhytMol has been developed as a database cum drug discovery platform that can be used to prioritize drug-like compounds. A number of experimentally verified anti-mycobacterial phytomolecules along with their experimental assay preparation and bioactivity details have been manually compiled from literature. Tools providing text searching, browsing and chemical similarity searching for phytomolecules have been integrated to utilize BioPhytMol as a drug discovery platform. The chemical structure similarity tool can be exploited to perform exact, substructure, superstructure and perfect search against various drug-like chemical libraries. This facility may be helpful to assign chemical classes and bioactivity to novel compounds. Users can also generate analogs for their target specific inhibitors. As of now the resource holds a total of 2582 entries including 188 plant families (comprised of 692 genera and 808 species) from global flora. In total, 633 purified compounds have been curated against 25 different target mycobacteria. These are reported along with their biological activity and experimental details as described below. The resource is available at http://ab-openlab.csir.res.in/biophytmol/.

## Construction and content

### Data source and structure

The PubMed literature database (http://www.ncbi.nlm.nih.gov/pubmed) and Google search (https://www.google.com/) were considered as major sources for collecting the plant derived anti-mycobacterial compounds and extracts. Initially, the relevant papers were downloaded using keywords such as ‘natural product’, ‘plant product’, ‘anti-tuberculosis’, ‘anti-mycobacterial’, ‘*Mycobacterium tuberculosis*’, ‘TB’ and ‘Mtb’. Papers with TB and natural product/plant product keywords in their abstract were further shortlisted. More than 150 research articles/reviews were used to retrieve anti-mycobacterial phytomolecules and plant extracts. A well-defined data structure (Additional file 1: Table S2) was followed to systematically extract, capture and store data manually for purified anti-mycobacterial phytomolecules and plant extracts. Wherever available, a PubChem ID (http://pubchem.ncbi.nlm.nih.gov/) has been assigned to purified phytomolecule. To maintain a common standard, wherever the activity was given in terms of μM, it was converted to μg/ml using GraphPad (http://www.graphpad.com/quickcalcs/Molarityform/). The structures unavailable in PubChem were drawn manually using MarvinSketch 5.11.4 software from ChemAxon (http://www.chemaxon.com/products/marvin/marvinsketch/) and were assigned a BioPhytMol ID.

### Data curation

The data was manually curated using a structured-wiki platform. To perform data quality check, a semi-automated procedure was followed based on a well-defined data structure as mentioned earlier. A set of dedicated curators also checked the data manually and well-curated data was finally stored in MySQL, an open-source relational database management system (RDBMS). For both data capture and curation, crowdsourcing through online platforms have been implemented. Each database entry is tagged with the contributor’s details.

### Platform architecture

BioPhytMol database has been developed using open source software LAMP (Linux-Apache-Mysql-PHP) server technology. PHP, HTML, JavaScript, AJAX and CSS technologies have been used to build the web interface. The whole software system runs on IBM SAS x3800 machine under Red Hat Enterprise Linux 5 environment using Apache httpd server.

## Utility

### User interface

The interface of BioPhytMol platform is customized to query the database with various searches and browse options. It is also bundled with various analysis tools to facilitate analysis of new compounds. In addition to this, the interface provides a link to update the database using a submission form. It also lists the database statistics along with physicochemical properties of the compounds. The data structure of the database and link to relevant TB and NP resources are also incorporated in BioPhytMol. The ‘BioPhytMol Help’ option has been used to explain how to use the platform. The homepage of the website has been shown in Additional file 1: Figure S1.

### Search tools

**Figure 1:**
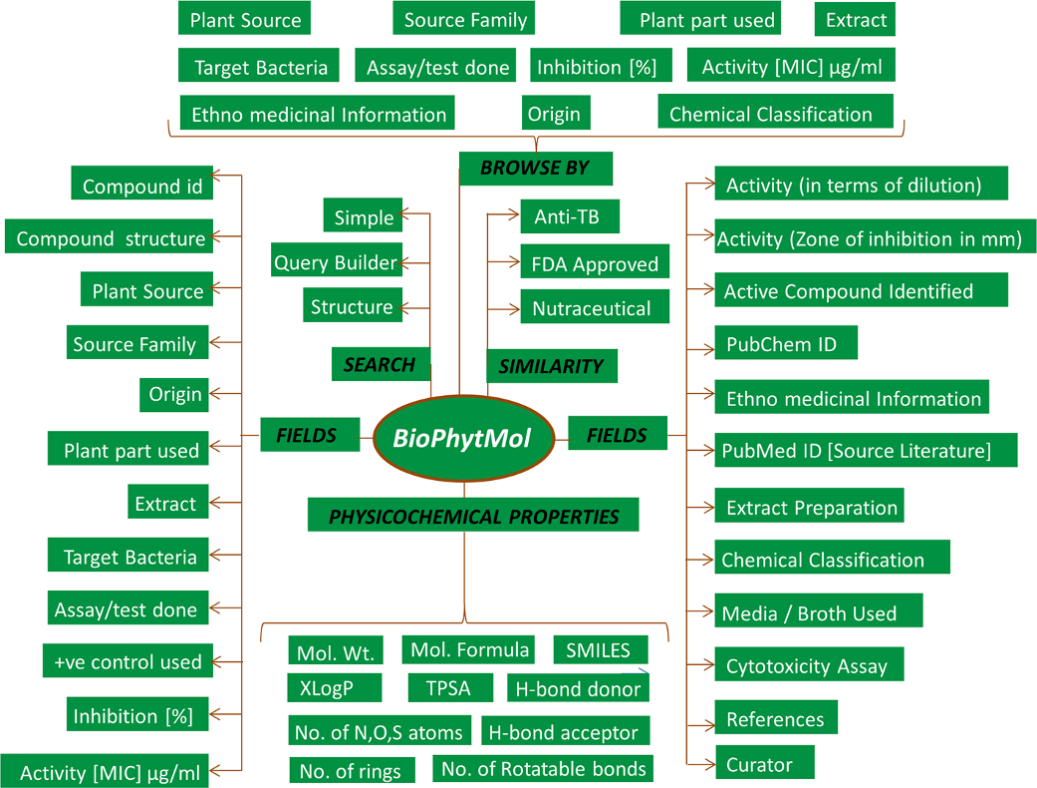
**BioPhytMol database architecture showing its various components with ‘Search’ options, ‘Browse by’ options and structural ‘similarity’ between BioPhytMol compounds and Anti-TB drugs, FDA approved small molecule drugs and Nutraceuticals.**

Three search options are provided on the web interface namely ‘Simple Search’, ‘Query Builder’ and ‘Structure Search’. Simple Search option allows users to perform text-based search on all/selected field of the database. Overall, the database contains 35 unique fields as shown in Figure 1.

Some of the major fields are (i) Compound ID (ii) Compound Structure (iii) Plant Source (iv) Source Family (v) Geographical Origin (vi) Plant Part Used (vii) Extract (vii) Target Bacteria (viii) Positive Control Used (ix) % inhibition (x) Activity [MIC] in μg/ml (xi) Active Compound Identified (xii) PubChem ID (xiii) Extract Preparation (xiv) Chemical Classification (xv) Media/Broth Used (xvi) Cytotoxicity Assay, etc. (See Additional file 1: Table S2 for details). User can display any or all fields of database for selected searched records.

The Query Builder option facilitates querying the database through complex queries with the help of logical operators such as AND & OR. This option helps to retrieve refined results, most relevant to user's query, such as compounds having molecular weight within a particular range, compounds having specific minimum inhibitory concentration (MIC) or following Lipinski’s rule of five [26], etc.

An extensive ‘Structure Search’ interface has been designed that allows users to find similar compounds from all/any of the drug-like classes by either drawing the structure, through SMILES format or uploading the chemical structure in SDF, MOL or MOL2 formats. In order to draw structures to be searched, a simple Java based applet - Java Molecular Editor (JME) (http://www.molinspiration.com/jme/) has been incorporated. A variety of structure search methods such as substructure, superstructure, exact and similarity based search have been provided. Users can perform structure based searches against BioPhytMol compounds (B_mols)/Anti-TB drugs (A_tb) and various DrugBank compound classes such as FDA approved small molecule drugs (FDA_small), FDA approved nutraceutical drugs (FDA_nutra), experimental, withdrawn and illicit drugs. The Structure Search is performed using jcsearch [27] that provides various searching options as mentioned above. Additional file 1: Figure S2 depicts the structure search tool provided in the resource.

### Browsing interface

The browsing interface provides an easy way to explore the BioPhytMol resource. Phytomolecules can be browsed using their physicochemical properties that include molecular weight, XLogP, polar surface area (PSA), hydrogen bond donor (HBD), hydrogen bond acceptor (HBA), number of Oxygen, Nitrogen and Sulphur atoms, number of rings and number of rotatable bonds. The major fields of database that can be browsed are shown in Figure 1. All the numerical fields can be sorted while browsing.

### Tools used for compound analysis and clustering

A set of tools and algorithms has been used for compound analysis in BioPhytMol. For detecting near neighbours (NNs), NNeib (version 5.12.0) (https://docs.chemaxon.com/display/jklustor/Jarvis-Patrick+clustering) was used. Clustering of B_mols was done using Jarp (version 6.0.2) from ChemAxon that follows variable length Jarvis-Patrick clustering algorithm (https://docs.chemaxon.com/display/jklustor/Jarvis-Patrick+clustering). Jarp uses the nearest neighbours information of the compounds in order to assign them into clusters based on dissimilarity threshold (i.e. 1-tanimoto coefficient). To compute the dissimilarity threshold, Jarp refers to the propriety chemical structure fingerprints calculated using ‘generatemd’ program of ChemAxon. These fingerprints are then used by NNeib program for calculating nearest neighbours using the dissimilarity threshold which is further used by the Jarp program of ChemAxon for assigning compounds into clusters. Detailed information can be found at: https://docs.chemaxon.com/display/jklustor/Jarvis-Patrick+clustering. A threshold of 0.15 is used as the dissimilarity threshold for clustering compounds. A total of 10 descriptors were calculated using PowerMV [28] descriptor calculator software namely molecular weight, XLogP, PSA, H-bond Donor, H-bond Acceptor, number of Rotatable Bond Count, number of rings, number of nitrogen atoms, number of oxygen atoms and number of sulphur atoms. Molecular formula of each compound was calculated using ChemAxon’s command line program ‘cxcalc’ (version 5.12.0) (https://docs.chemaxon.com/display/CALCPLUGS/cxcalc+command+line+tool). To check for compound’s drug-likeness, a variety of filters were applied using DruLiTo (http://www.niper.gov.in/pi_dev_tools/DruLiToWeb/DruLiTo_index.html).

### Visualization tools

BioPhytMol facilitates the users to display 2-D and 3-D structure of molecules using images and Jmol (http://www.jmol.org/), respectively. Wordle (http://www.wordle.net/) (developed by Jonathan Feinberg), was used to develop the tag cloud of various chemical classes of B_mols. For the first time, we have customized CIRCOS [29] (http://circos.ca/) to visualize multiple chemical properties with biological activity and obtain clusters for near neighbour analysis. CIRCOS is originally designed for visualization of genomic data in the form of circular layout (graph) and hence the configuration and data files represent data in context of chromosome positions. The karyotype file in CIRCOS package is used to define the data points on this circular graph. The same concept has been translated to visualization of chemical and biological data associated with B_mols. In this study, 633 data points were defined (bands) with each band having a width of 1000. This baseline is then used to highlight cyclic and aliphatic compounds. Further customization needs appropriate modifications to generate corresponding histogram files. For better visualization of nature of relationship amongst members of clusters a compound network diagram (CND) was made using Cytoscape (http://www.cytoscape.org/).

### BioPhytMol statistics

At present, BioPhytMol resource holds a total of 2582 entries including 188 plant families (comprised of 692 genera and 808 species) from global flora. In total, 633 purified compounds have been curated against 25 different target mycobacteria. These B_mols are reported along with their biological activity and experimental details including the assay performed, target bacteria, positive control, etc. Of the 633 compounds, 125 (20%) listed in BioPhytMol have more than one MIC reported against them. The remaining 495 (~78%) compounds have single MIC values and 13 B_mols have no MIC data. The geographical distribution of plants shows that majority of anti-mycobacterial phytomolecules/plant extracts are from Indian origin followed by Africa, Mexico, Peru and Malaysia (http://ab-openlab.csir.res.in/biophytmol/browse_origin.php?f=origin). Additional file 1: Figure S3 shows the distribution of plants among top 35 plant families present in the resource with Asteraceae, Fabaceae and Rutaceae having more than hundred plants each. ‘Asteraceae’ has the highest number of entries (215) in the resource. *Helichrysum melanacme*, a plant from Asteraceae family, showed the lowest MIC against Mtb at 0.05 μg/ml. From the same family, *Chrysanthemum morifolium* produces the highest number of anti-mycobacterial compounds with varying range of MICs (4–64 μg/ml). For example, 3-Epilupeol was active against Mtb at 4 μg/ml, while Calenduladiol was active against Mtb at 64 μg/ml. From Additional file 1: Figure S3, it is also observed that there are families which contain less than 25 plants. *Cordia globifera* from ‘Boraginaceae’ family was reported active against H37Ra strain of Mtb at 1.5 μg/ml. It may be noted that highly potent anti-mycobacterial compounds may be present in other plant families too. Hence, underrepresented families in our database may be further explored to find new plants with anti-mycobacterial properties.

### Diversity in B_mols

Clustering of 633 B_mols produced a total of 423 clusters of which 318 clusters (50.24% of B_mols) were singletons while remaining 105 clusters (49.76% of B_mols) were non-singletons (see Additional file 1: Figure S4). This indicates that BioPhytMol library is chemically diverse. For better visualization of the nature of relationship amongst members of the clusters, the CND generated using Cytoscape may be seen at http://ab-openlab.csir.res.in/biophytmol/circos.php. The nodes in the CND are color coded with blue color representing active compounds, red representing inactive compounds and yellow is where MIC data is unavailable. A threshold MIC of > =200 μg/ml is used for segregating active B_mols and inactive B_mols. This threshold classifies 633 B_mols into 582 active compounds (MIC < 200 μg/ml), 38 inactive compounds while MIC values were unavailable for 13 B_mols. As can be seen from the CND (http://ab-openlab.csir.res.in/biophytmol/circos.php), only 14 inactive compounds cluster with 301 active compounds in 105 clusters. A table with compounds in each cluster is provided along with the CND for better interpretation of the data (http://ab-openlab.csir.res.in/biophytmol/circos.php#biophyt_clusters).

### CIRCOS visualization and analysis of chemical and biological properties

**Figure 2:**
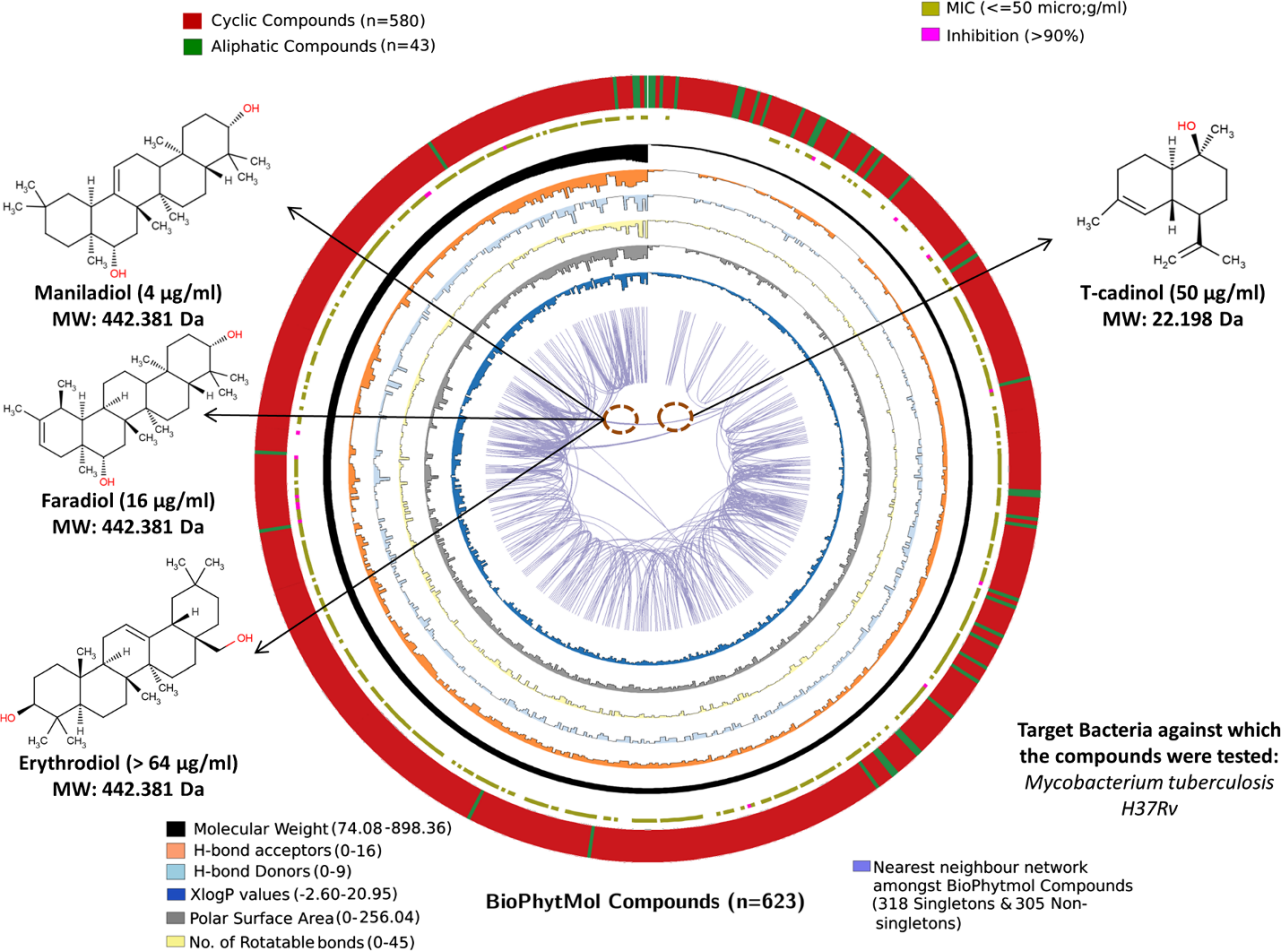
**Overall distribution of chemical, biological and physicochemical properties of 623 BioPhytMol phytomolecules.** The outermost circle represents the broad chemical structure of compounds i.e., they are cyclic or aliphatic. The next ring depicts the biological activity of the compounds in terms of MIC ≤ 50 μg/ml and mycobacterial growth % inhibition > 90. The subsequent rings represent six graphs of calculated physicochemical properties. All the compounds are sorted in the increasing order of their molecular weight. The innermost graph is the representation of near neighbours (NN) amongst B_mols. The four compounds: T-cadinol, Maniladiol, Faradiol and Erythrodiol are nearest neighbours with difference in molecular weight between T-cadinol and the rest of the three compounds. Important structural differences may be the reason behind different MICs.

As discussed, BioPhytMol resource includes chemical details and biological data for the phytomolecules. The chemical details include structure, physicochemical properties and near neighbours. The biological data includes mycobacterial growth % inhibition and MIC of these compounds. Attempt has been made to represent this multidimensional data through circular graphs using CIRCOS. The overall distribution of important physicochemical properties of B_mols with biological data is depicted in Figure 2.

The outermost ring represents the broad chemical structures of the compounds indicating that majority of the compounds are cyclic in nature. The next ring depicts the biological activity of the compounds with MIC ≤ 50 μg/ml and % inhibition > 90. The subsequent rings represent six graphs of calculated physicochemical properties namely molecular weight, hydrogen bond acceptor, hydrogen bond donor, XLogP, polar surface area and number of rotatable bonds. All 633 compounds were sorted in the increasing order of their molecular weight. Compounds with molecular weight > 900 Daltons (Da) (10 compounds) were removed subjectively and are not represented in the CIRCOS plot and thus, only 623 compounds have been used to plot Figure 2. A list of another six CIRCOS plots sorted on the basis of six physicochemical properties, namely, molecular weight, XLogP, number of hydrogen bond donors, number of hydrogen bond acceptors, polar surface area and number of rotatable bonds, are provided on the BioPhytMol platform (http://ab-openlab.csir.res.in/biophytmol/circos.php#biophyt_circos). Each physicochemical property was sorted to retrieve six different CIRCOS plots. Outliers were removed from sorted physicochemical property data before generating the plots. The following formula was used to remove the outliers:

$$\left[Q_{1}-k\left(Q_{3}-Q_{1}\right),Q_{3}+k\left(Q_{3}-Q_{1}\right)\right]$$

Where Q_1_ and Q_3_ are the first and third quartile respectively and *k* is the constant (default value of *k* =1.5). The above formula gives a range in the data. Any value outside this range in the data is taken as an outlier.

The innermost graph in Figure 2 (sorted on molecular weight) represents the near neighbours amongst B_mols. It is interesting to observe in some cases that compounds that span different molecular weight range are near neighbours. For example, T-cadinol (22.198 Da) has three near neighbours: Maniladiol (442.381 Da), Faradiol (442.381 Da) and Erythrodiol (442.381 Da). Presence of certain structural features in ‘Maniladiol’ may be responsible for its better anti-mycobacterial activity against Mtb H37Rv (4 μg/ml) as compared to T-cadinol, Faradiol and Erythrodiol (16–64 μg/ml). Hence, this approach is useful in finding out pharmacophoric features that may be important in contributing to the anti-mycobacterial property of a particular compound. At least twenty cases have been observed where the nearest neighbours have shown large difference in their MIC values. For example, N-trans-Feruloyltyramine (Additional file 1: Figure S5 A) and Feruloyltyramine (Additional file 1: Figure S5 B) were active against Mtb H37Rv at an MIC of 128 μg/ml and 1.6 μg/ml respectively. An additional methoxy group (−OCH3) in Feruloyltyramine (as depicted in Additional file 1: Figure S5 C by dotted circle) may be responsible for defining the pharmacophoric feature of the compound.

### Physicochemical distribution of B_mols with respect to FDA_small, FDA_Nutra and A_tb

To estimate the oral bioavailability and drug-likeness of B_mols, these were filtered on the basis of physicochemical properties as suggested by Lipinski and Veber [30]. Also, physicochemical distribution, as followed by ≥ 70% FDA approved drugs, was taken into account simultaneously. Of 633 phytomolecules, 232 passed these filters (http://ab-openlab.csir.res.in/biophytmol/extract_fda_oral.php). Physicochemical properties of the entire B_mols have been compared with those of FDA_small, FDA_nutra and A_tb. It was observed that the highest peak for molecular weight of B_mols lie within 201–300 (Additional file 1: Figure S6 A). This is in accordance with both Lipinski rule of 5 and Lipinski rule of 3 for antibacterial compounds [31].

Number of HBD for B_mols showed highest peak in the range of 1–3 (Additional file 1: Figure S6 B). Similar trend was observed for rest of the drug classes. As for HBA, majority B_mols, A_tb and FDA_nutra lied in the range of 1–3 (Additional file 1: Figure S6 C). In the case of XLogP, a high peak was observed in the case of B_mols, FDA_small and A_tb which was in the range of 4–7 (Additional file 1: Figure S6 D). PSA and number of rotatable bonds are good physicochemical property to predict oral bioavailability of a drug. In past, it has been observed that compounds having PSA ≤ 140 Å^2^ and number of rotatable bonds ≤ 10 possess higher probability of good oral bioavailability [30]. The highest peak of B_mols for PSA and number of rotatable bonds lie between 51–100 Å^2^ (Additional file 1: Figure S6 E) and 1–3 (Additional file 1: Figure S6 F), respectively.

### Structural similarity of B_mols with existing drugs

Using NNs search, B_mols similar to A_tb, FDA_nutra and FDA_small have been identified. The result of this NNs search is reported in two sections under 'Similarity' option: (i) One to one; (ii) One to many. In ‘One to one’, B_mol is shown paired with its similar drug and calculated dissimilarity threshold value for that pair, e.g., four B_mols showed similarity with Amikacin (a second-line anti-TB drug) and six B_mols showed similarity with Clarithromycin (third-line anti-TB drug) at 0.4 dissimilarity threshold (http://ab-openlab.csir.res.in/biophytmol/str_stats_man.php). These B_mols may first be evaluated for presence of potential toxicophores. Based on this prioritization, they may be further experimentally tested for anti-TB activity *in-vitro* and *in-vivo*.

Owing to wide range of indications covered by nutraceuticals such as less toxicity, physiological health benefits and good oral bioavailability, they are gaining popularity as promising drug candidates [32]. NNs search of B_mols against FDA_nutra resulted into identification of four phytomolecules (http://ab-openlab.csir.res.in/biophytmol/fda_nutra.php). For example, Stigmasta-4-22-dien-3-one (BioPhytMol ID - 2205) active against Mtb at < 2 μg/ml, is a phytomolecule (extracted from leaf of *Morinda citrifolia L.*) that showed structural similarity with 19-norandrostenedione (DrugBank ID - DB01434), once marketed as a dietary supplement. Overall, there are 10, 4 and 16 B_mols similar to 2 A_tb, 4 FDA_nutra and 15 FDA_small, respectively (see Additional file 1: Table S3).

### Drug-likeness of B_mols

**Figure 3:**
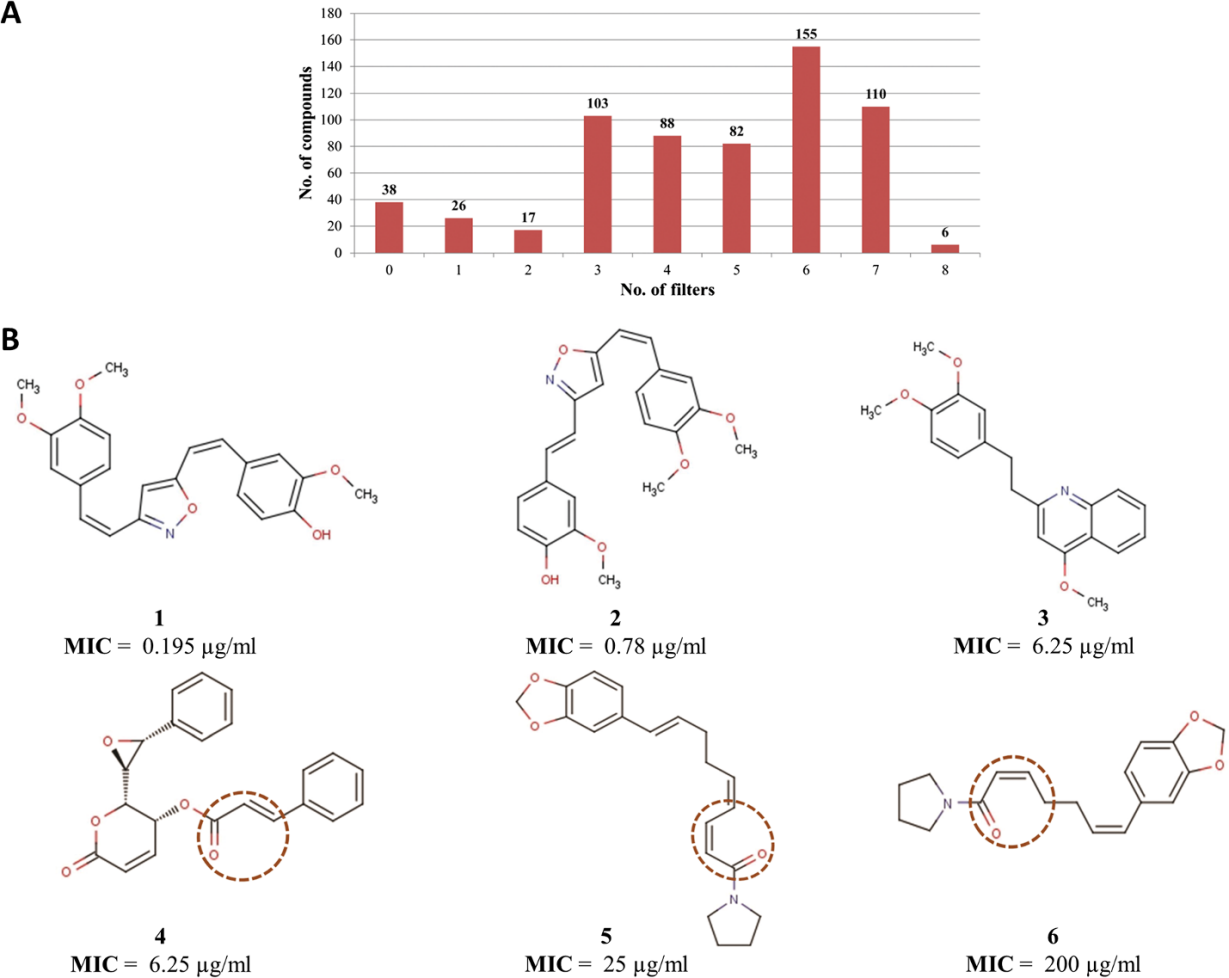
**Drug-likeness study of BioPhytMol compounds. (A)** Compound distribution graph based on the number of compounds screened against DruLiTo (Drug-likeness Tool) filter with 6 compounds passing all 8 filters. **(B)** Structure of BioPhytMol compounds crossing all the filters of DruLiTo: **(1)** 4-((Z)-2-(3-(3,4-dimethoxystyryl)isoxazol-5-yl)vinyl)-2-methoxyphenol **(2)** 4-((E)-2-(5-(3,4-dimethoxystyryl)isoxazol-3-yl)vinyl)-2-methoxyphenol **(3)** 2-(3,4-dimethoxyphenethyl)-4-methoxyquinoline **(4)** (2S,3R)-6-oxo-2-((2S,3R)-3-phenyloxiran-2-yl)-3,6-dihydro-2H-pyran-3-yl cinnamate **(5)** (2Z,4Z,8E)-9-(benzo[d][1,3]dioxol-5-yl)-1-(pyrrolidin-1-yl)nona-2,4,8-trien-1-one **(6)** (2Z,6Z)-7-(benzo[d][1,3]dioxol-5-yl)-1-(pyrrolidin-1-yl)hepta-2,6-dien-1-one. The dotted circle shows the presence of a Michael acceptor group (toxicophore).

To prioritize drug-like phytomolecules, DruLiTo software was used to screen molecules based on eight filters namely Lipinski's rule, MDDR-like rule, Veber rule, Ghose filter, BBB rule, CMC-50 like rule, weighted and unweighted Quantitative Estimate of Drug-likeness [33]-[35]. As evident from Figure 3A, six of 633 compounds passed all eight filters.

It was also noticed that out of these six compounds, three compounds i.e., Figure 3B 4–6, had a Michael acceptor moiety (double bond next to a carbonyl group, highlighted by dotted circle) (http://bioserv.rpbs.univ-paris-diderot.fr/FAF-Drugs/). The presence of this functional group makes the compound ‘non-leadlike’ which gives false positive results in biochemical assays. Hence, filters for drug-likeness are not enough to state that the compounds will be successful in further drug discovery stages, but identification of such ‘warhead agents’, ‘frequent hitter’ and ‘promiscuous inhibitors’ are important to evaluate the chances of success for a particular scaffold in a drug discovery process [36].

In a recent study published by GSK, 177 anti-TB hits with low *in-vitro* cytotoxicity were reported [37]. On comparison of 633 B_mols with 177 hits, 20 B_mols showed similarity with two GSK compounds. Of these 20 B_mols, 6 were similar to GSK compound GSK146660A and 14 were similar to GSK1996236A. These compounds are predicted to act via Rv0458, Rv0548c, Rv1747, Rv2971 and Rv3170 [38]. Additional file 1: Table S4 can be seen for complete details.

This approach could be used to identify putative targets for other B_mols/novel compounds where target information is missing. Another freely available target identification tool published recently includes TB Mobile 2.0 which identifies potential targets based on molecular similarity [39]. Alternatively, one can also assess the target specific inhibitors for their activity in whole cell screens.

### Natural products ontology and chemical classification

Presence of a universal ontology is necessary for global understanding of NP data. National Center for Biomedical Ontology (NCBO) has developed one such ontology for NPs- the Natural products Ontology (NATPRO) (http://bioportal.bioontology.org/ontologies/NATPRO). Of 35 BioPhytMol metadata, 12 mapped to various levels in NATPRO metadata (see Additional file 1: Table S5). However, the chemical classification of NATPRO was not sufficient to broadly classify the compounds.

Classification of chemical compounds aids the evaluation and abstraction of compound properties. In addition, it may enable new ways of knowledge discovery for example by extracting relationships between compound structure and its properties, traditionally known as structure-activity relationships (SAR) or structure property relationships (SPR). The basic idea behind this classification is to be able to connect the properties of the compounds with their biological activity. The compounds have been classified broadly into three levels. In the primary level, the compounds were classified into aromatic (312), alicyclic (274) and aliphatic (46) compounds. One compound was classified as a mixture. At the secondary level, each of the three classes are further divided based on presence of specific groups (e.g., Aryl group), no. of carbon-carbon bonds (e.g., alkane, alkene or alkyne) and no. of cycles. In the tertiary level, the classification is based on functional groups like alcohol, ketone, aldehyde, ester, etc. and broad classes like steroid, terpene, triterpene, sesquiterpene, sugar, furan, etc. Additional file 1: Figure S7 shows an example of an aromatic compound containing ether, phenol and ketone as functional groups and it belongs to two classes i.e. chalcone and sugar. In the browse page of BioPhytMol website, prominent classes in BioPhytMol have been shown as a tag-cloud (http://ab-openlab.csir.res.in/biophytmol/browse.php). From the tag-cloud, it can be seen that majority of the B_mols are aromatic (312) in nature with many compounds having phenol (176 compounds) and ether (161) as functional groups. The complete chemical classification of B_mols can be found from the link given below: http://ab-openlab.csir.res.in/biophytmol/browse.php?f=chem_class.

## Discussion

BioPhytMol is a drug-discovery community resource with a database of anti-mycobacterial phytomolecules and plant extracts with analysis tools integrated into a web-application. The database is based on extensive literature search and curation and is the only searchable resource on anti-mycobacterial phytomolecules. In addition to the data on plant extracts or the purified active ingredients, the structure, bioactivity and experimental details are also captured systematically through a well-defined data structure. This platform is designed to perform analysis based both on chemical structure and biological properties which is the primer for any early stage drug discovery process. To facilitate structure-based analysis, BioPhytMol has been equipped with structure-based search options, which may be used to compare any new compound with the BioPhytMol database, as also with anti-TB drugs and compounds reported in DrugBank in various categories. These searches are expected to reveal shared/distinct structural features of the new and known anti-TB drugs with that of other drug-like libraries including small molecules and nutraceuticals. It is interesting to observe that there are cases where a compound has been reported to show activity against multiple species of mycobacteria. This feature of database may help in identification of the structural features responsible for highly selective or broad-spectrum activity of phytomolecules against different mycobacterial strains. Similarly, the data and the analysis tools also enable identification of structural analogs of reported anti-TB compounds and inhibitors of specific drug targets in Mtb.

A revised ontology and chemical classification is also proposed as part of systematic analysis of the compound library. This is important for data-interoperability and shared knowledgebase. The revised chemical classification, though not extensive, allows the chemists to search for compounds with specific chemical features like aromaticity or functional groups like ketone or classes like specific sugar moieties, thus, making the platform useful for researchers from multiple disciplines.

A highlight of the study is the representation of multidimensional data through circular graphs that allow interpretation of various chemical properties with respect to each other and with bioactivity data. CIRCOS has been used to generate these graphs. CIRCOS is a very popular tool among genomics and genetics community to represent the physical map and annotation of circular genomes. For this study, CIRCOS has been customized to represent the chemical and biological properties of the compound library. The same approach can be used to represent any set of properties and is expected to aid in interpretation of multi-dimensional data.

The real success of a database lies in its regular updation. Database updation and curation is an on-going activity. To ensure that BioPhytMol is regularly updated, a structured-wiki platform has been designed. This platform allows community curation of new datasets as well as modification of the existing one for any ambiguities. The submitters are duly acknowledged for their contribution as each annotation or entry is author and time stamped. The curators should register through the OSDD portal and obtain Sysborg OpenID. This ID can then be used to update the database through online form. However, database changes are committed only when the submission is quality checked for correctness and format. Efforts are ongoing to extend the search base of BioPhytMol as well as to include batch query options where multiple structures can be searched using the similarity search tool for their corresponding similar structures.

## Conclusions

BioPhytMol is a drug-discovery community resource with a database of anti-mycobacterial phytomolecules and plant extracts. This a unique resource that encapsulates important information such as their bioactivity values (MIC), target bacteria, percentage inhibition of mycobacterial growth and most importantly two dimensional as well as three dimensional structures for purified anti-mycobacterial phytomolecules. Structure based searching and comparison against various drug classes aids to the features of platform to speed up the anti-mycobacterial drug discovery.

## Availability and requirements

BioPhytMol is freely accessible at http://ab-openlab.csir.res.in/biophytmol/. To access BioPhytMol, World Wide Web is a pre-requisite. To access all features of BioPhytMol to its optimum level, JavaScript and Java Runtime Environment (JRE) plugin must be enabled. The raw data may be accessed at the electronic lab notebook available at http://sysborg2.osdd.net/group/sysborgtb/lab-notebook-details/-/labnotebooks/show/6546.

## Authors’ contributions

BD, GJJ and TK compiled data from literature. AS and PD curated the data and updated the fields wherever data was incomplete. MS curated the chemical structures. AS developed the database, web-based tools and the interface of BioPhytMol with inputs from PD. AB, AS, PD, NKR performed compound analysis. Chemical classification was done by MS and AB. NKR prepared visualization plots, CIRCOS and CND. AS, PD and AB drafted the manuscript. AB conceived and designed the project and refined the manuscript. AB defined the SOP for data collection and also the data structure. The manuscript is read and approved by all authors.

## Additional file

## Electronic supplementary material

Additional file 1: Figures: screenshots of home page and structure search form of BioPhytMol; distribution of plants among top 35 plant families; near neighbours present amongst BioPhytMol compounds; compounds with small structural difference but huge MIC difference; distribution of physicochemical properties of BioPhytMol compounds with respect to different classes of drugs; example of proposed simplified ontology for chemical classification. Tables: existing anti-TB drugs classified according to their drug class and differentiated as per their origin (natural or synthetic); data structure and definition of data fields of BioPhytMol; BioPhytMol compounds (B_mols) showing similarity to various drug classes - anti-TB drugs (A_tb), FDA approved nutraceutical drugs (FDA_nutra) and FDA approved small molecule drugs (FDA_small); twenty B_mols near neighbours of two GSK compounds (GSK146660A and GSK1996236A) with known target information; BioPhytMol database fields mapped on the NCBO Natural products ontology (NATPRO). (DOC 14 MB)

Below are the links to the authors’ original submitted files for images.

Authors’ original file for figure 1

Authors’ original file for figure 2

Authors’ original file for figure 3

Authors’ original file for figure 4

## Abbreviations

TB: Tuberculosis
Mtb: *Mycobacterium tuberculosis*
MDR: Multi-drug resistant
XDR: Extensively drug resistant
TDR: Totally drug-resistant
FDA: Food and Drug Administration
NP: Natural product
UNPD: Universal Natural Products Database
LAMP: Linux-Apache-Mysql-PHP
MIC: Minimum inhibitory concentration
JME: Java Molecular Editor
B_mols: BioPhytMol compounds
A_tb: Anti-TB drugs
FDA_small: FDA approved small molecule drugs
FDA_nutra: FDA approved nutraceutical drugs
PSA: Polar surface area
HBD: Hydrogen bond donor
HBA: Hydrogen bond acceptor
NNs: Near neighbours
CND: Compound network diagram
Da: Daltons
NCBO: National Center for Biomedical Ontology
NATPRO: Natural products Ontology
SAR: Structure-activity relationships
SPR: Structure property relationships
