# Supplementary material for: BioPhytMol: a drug discovery community resource on anti-mycobacterial phytomolecules and plant extracts
Source: J Cheminform. 2014 Oct 11;6:46. doi: 10.1186/s13321-014-0046-2 (PMC4206768; doi:10.1186/s13321-014-0046-2)
Supplement: Supplementary file 1 — Additional file 1: Figures: screenshots of home page and structure search form of BioPhytMol; distribution of plants among top 35 plant families; near neighbours present amongst BioPhytMol compounds; compounds with small structural difference but huge MIC difference; distribution of physicochemical properties of BioPhytMol compounds with respect to different classes of drugs; example of proposed simplified ontology for chemical classification. Tables: existing anti-TB drugs classified according to their drug class and differentiated as per their origin (natural or synthetic); data structure and definition of data fields of BioPhytMol; BioPhytMol compounds (B_mols) showing similarity to various drug classes - anti-TB drugs (A_tb), FDA approved nutraceutical drugs (FDA_nutra) and FDA approved small molecule drugs (FDA_small); twenty B_mols near neighbours of two GSK compounds (GSK146660A and GSK1996236A) with known target information; BioPhytMol database fields mapped on the NCBO Natural products ontology (NATPRO). (DOC 14 MB) [file 13321_2014_46_MOESM1_ESM.doc]

# Supporting Information BioPhytMol: a drug discovery community resource on anti-mycobacterial phytomolecules and plant extracts

Arun Sharma^1,†^
Email: bioinfo.arun@gmail.com

Prasun Dutta^1,†^
Email: prasundutta87@gmail.com

Maneesh Sharma^4^
Email: maneeshsharma77@gmail.com

Neeraj Kumar Rajput^1^
Email: nk.rajput812@gmail.com

Bhavna Dodiya^2^
Email: bhavnadodia@gmail.com

John J Georrge^3,5^
Email: johnjgeorrge@gmail.com

Trupti Kholia^3^
Email: kholia.trupti@gmail.com

OSDD Consortium
Email: info@osdd.net

Anshu Bhardwaj^1*^
^*^ Corresponding author
Email: anshub@osdd.net

^1^ Open Source Drug Discovery (OSDD) Unit, Council of Scientific and Industrial Research, New Delhi, India

^2^ Department of Applied mathematics and Bioinformatics, Faculty of technology and engineering, The Maharaja Sayajirao University of Baroda, Vadodara, Gujarat, India

^3^ Department of Bioinformatics, Christ College, Rajkot, Gujarat, India

^4^ St. Stephens College, University of Delhi, New Delhi, India

^5^ Department of Biochemistry and Molecular Biology, University Clinic of Bonn (UKB), University of Bonn, Bonn, Germany

^†^ Equal contributors.

**
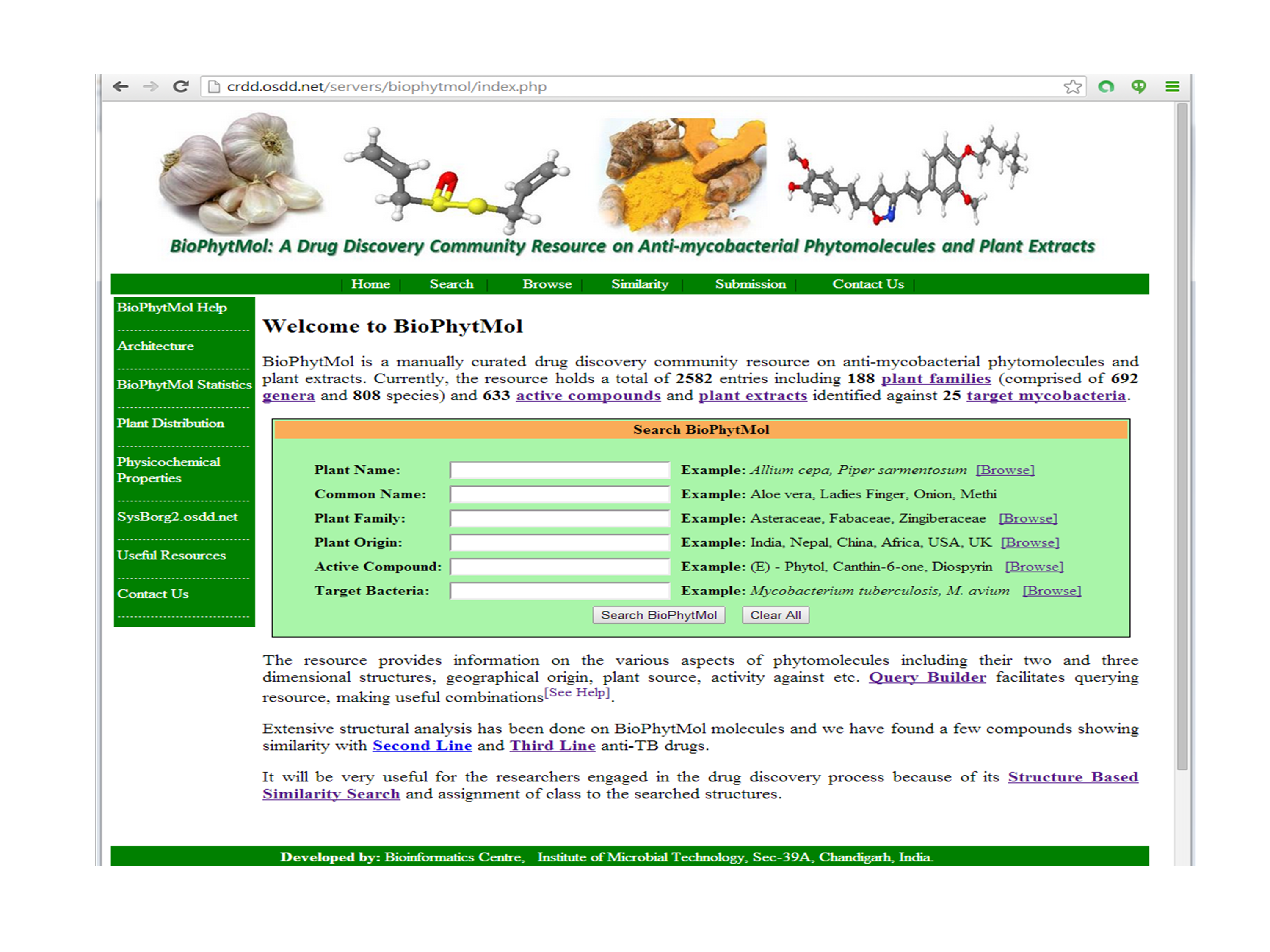
**

**Figure S1.** Homepage of BioPhytMol with menu bar at the top: The ‘Search’ option allows the user to search the resource using 'Simple Search'/'Query Builder'/'Structure Search'. The anti-mycobacterial phytochemicals/plant extracts can be explored on various database fields using the ‘Browse’ option. ‘Similarity’ option shows the results of nearest neighbour search of BioPhytMol compounds (B_mols) with other drug-like classes. ‘Submission’ permits the users to update the database entries. On the left panel, the user can find information on the database architecture of the resource, statistical and physicochemical information of B_mols and information on other natural products and TB database/resource information can be found from ‘Useful Resources’. BioPhytMol is available at http://ab-openlab.csir.res.in/biophytmol/.

**
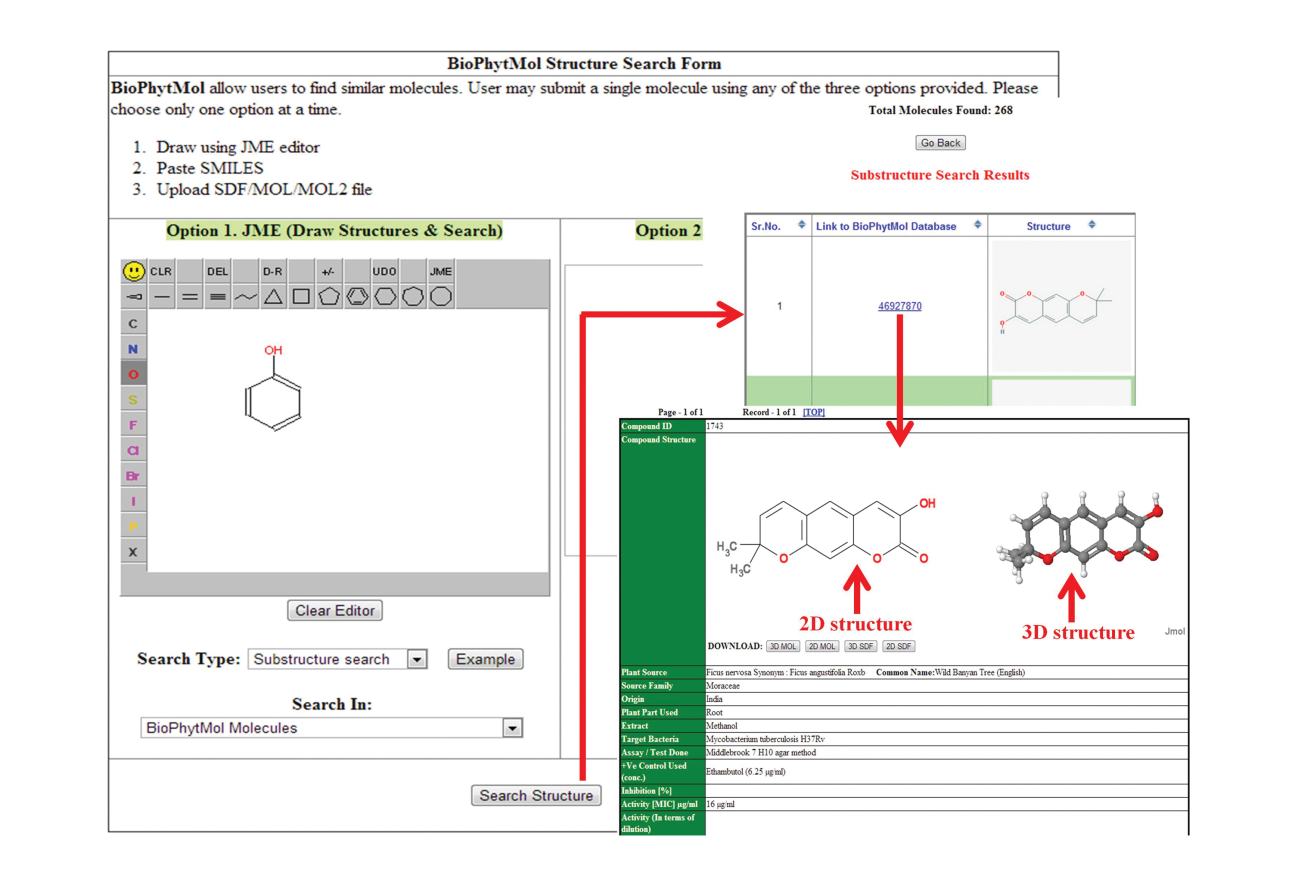
**

Figure S2. Structure Search Form of BioPhytMol: This allows the user to search the resource for compounds either by drawing the chemical structure, through the SMILES notation of the compound or by uploading the compound in SDF, MOL or MOL2 format. This particular example demonstrates how the user can draw the chemical structure and perform a substructure search against BioPhytMol compounds.

**
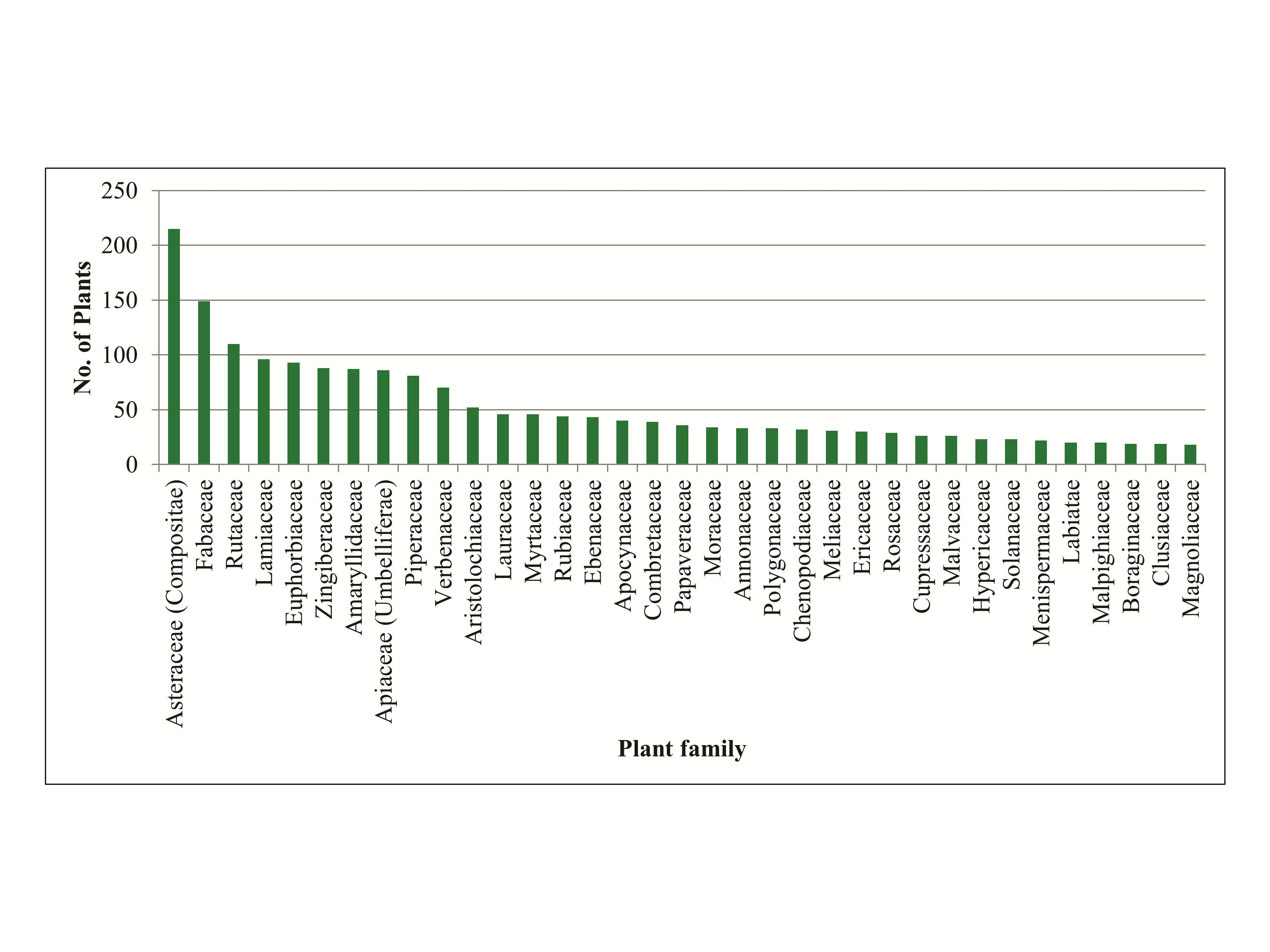
**

Figure S3. Distribution of top 35 families present in BioPhytMol database

**
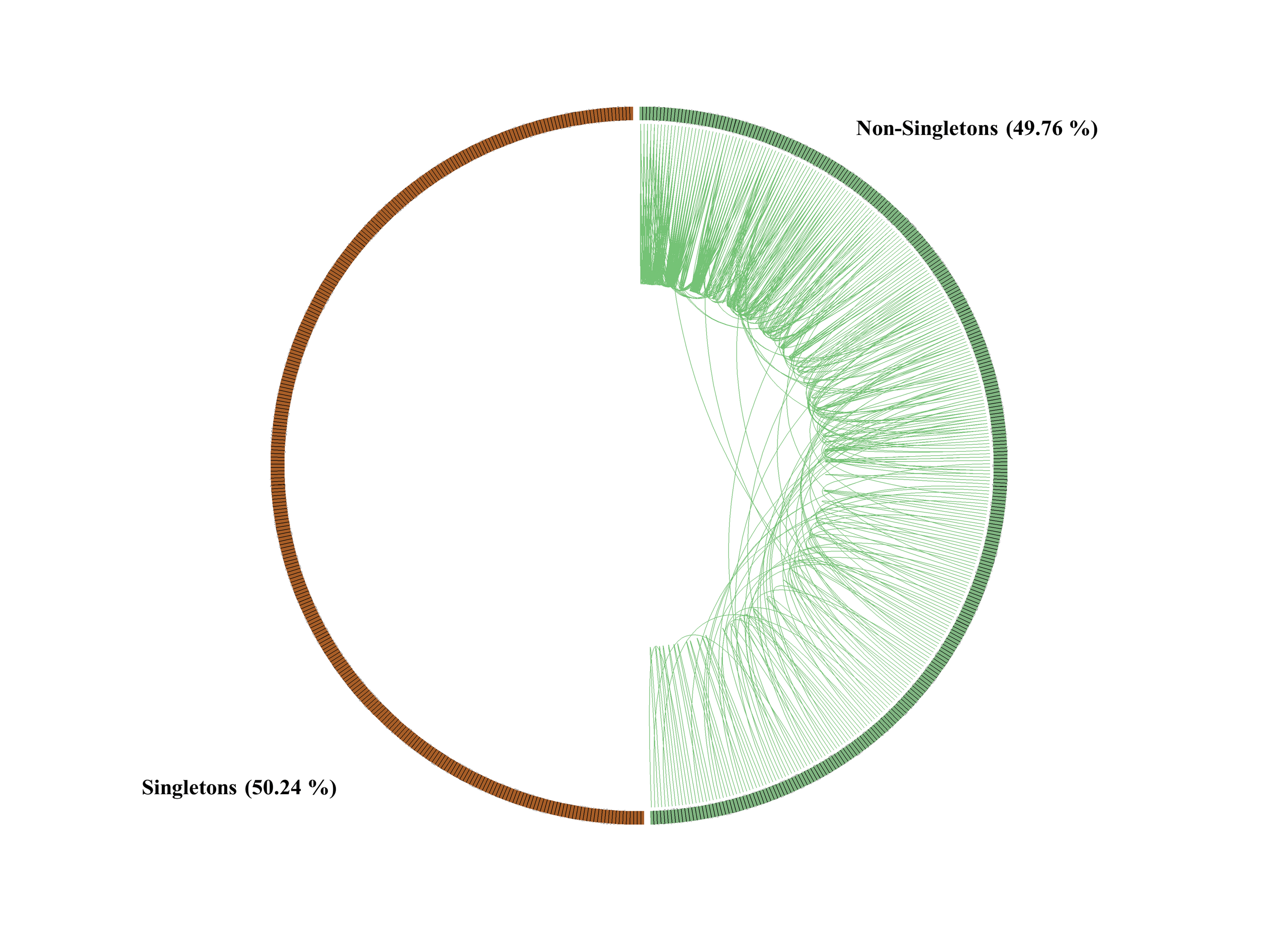
**

**Figure S4.** Diagrammatic representation of near neighbours present amongst BioPhytMol compounds using CIRCOS. 50.24 % of the compounds are singletons and 49.76 % are non-singletons (have near neighbours)

**
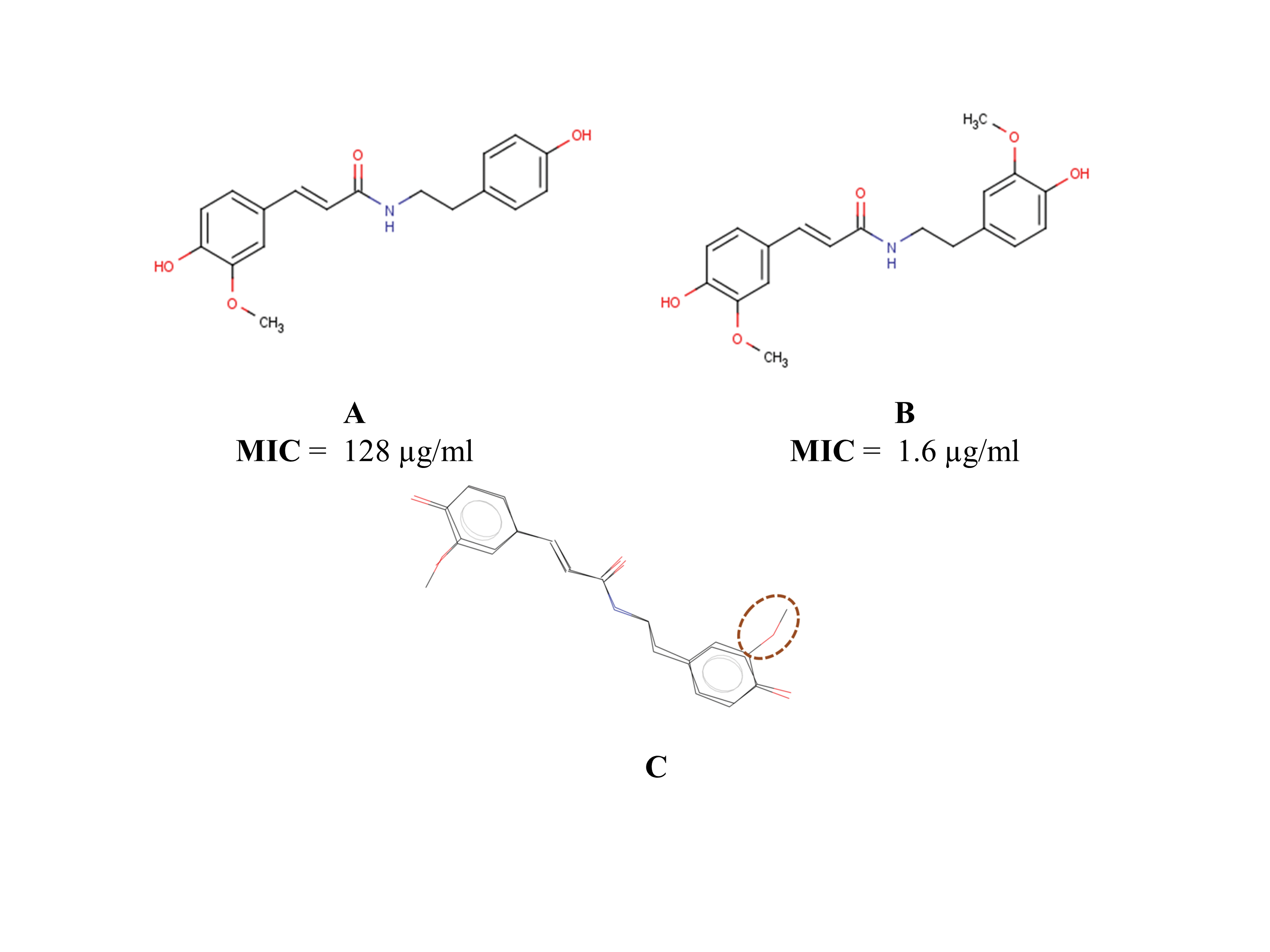
**

**Figure S5.** **(A)** N-trans-Feruloyltyramine, **(B)** Feruloyltyramine, **(C)** Superimposition of N-trans-Feruloyltyramine and Feruloyltyramine showing the presence of additional methoxy group on Feruloyltyramine (dotted circle). Both compounds (A and B) have been reported as active against Mtb H37Rv at MIC of 128 µg/ml and 1.6 µg/ml, respectively. The additional methoxy group (-OCH3) in Feruloyltyramine may be responsible for defining the pharmacophoric features of the compound.

**
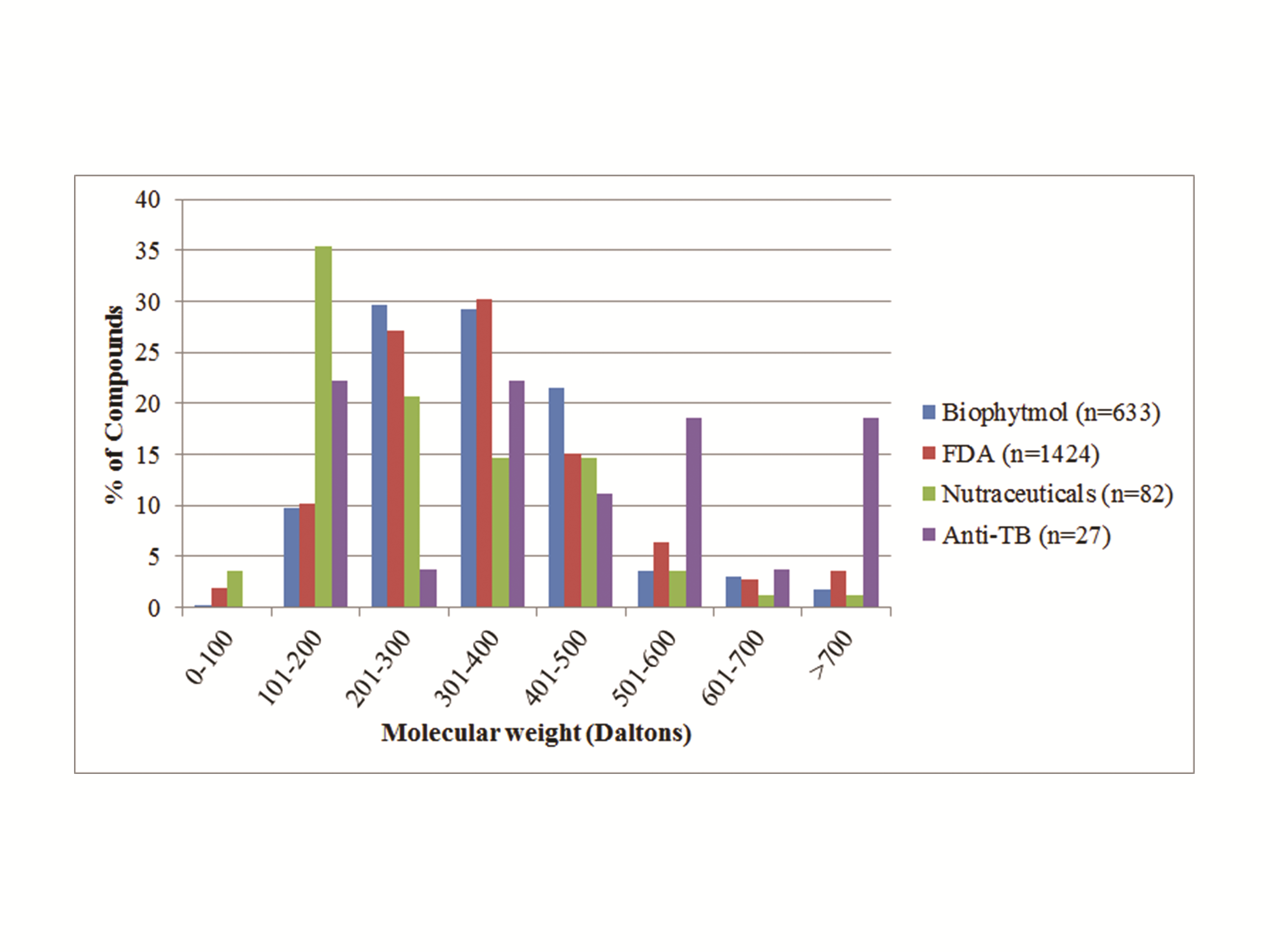

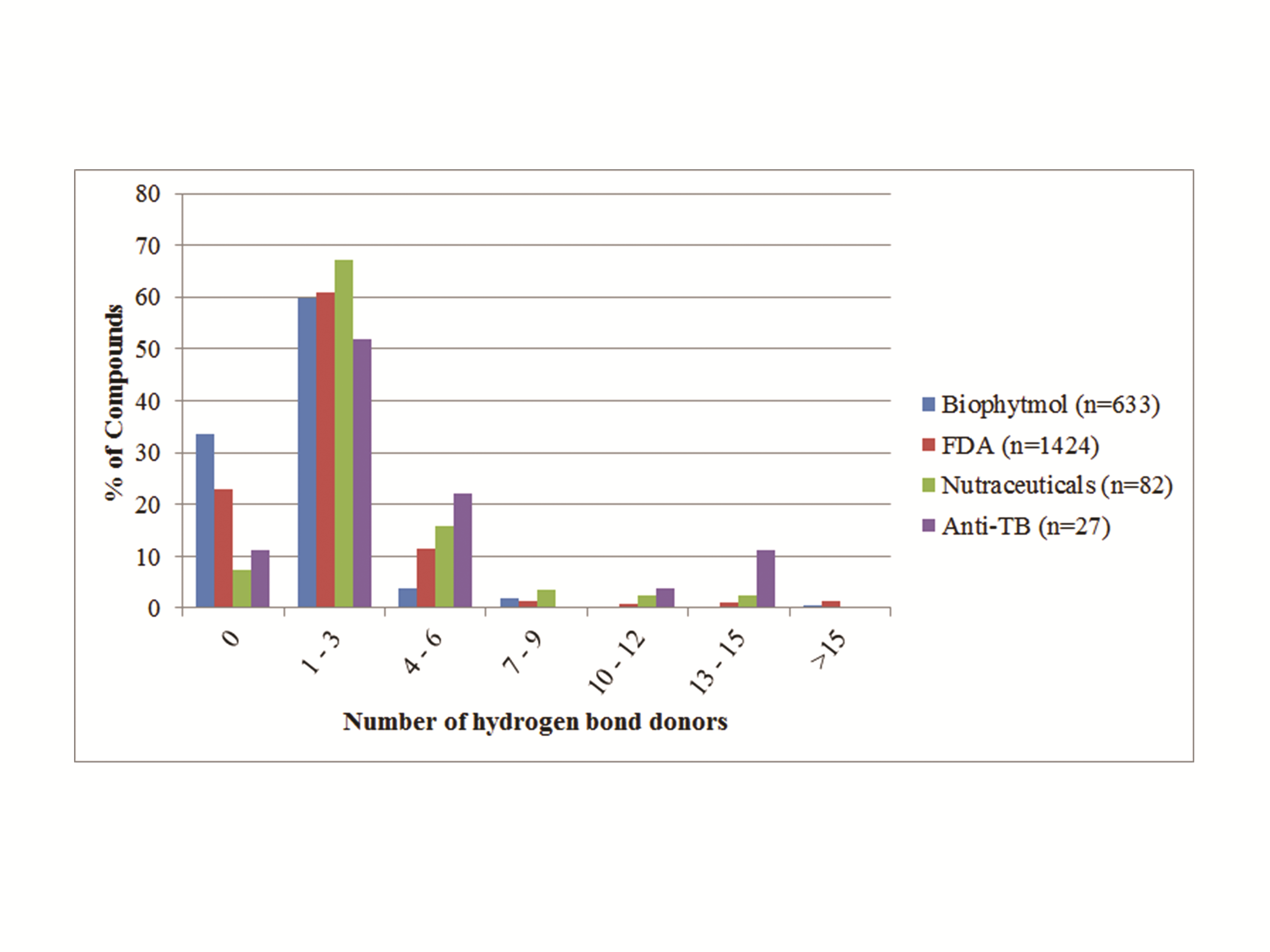

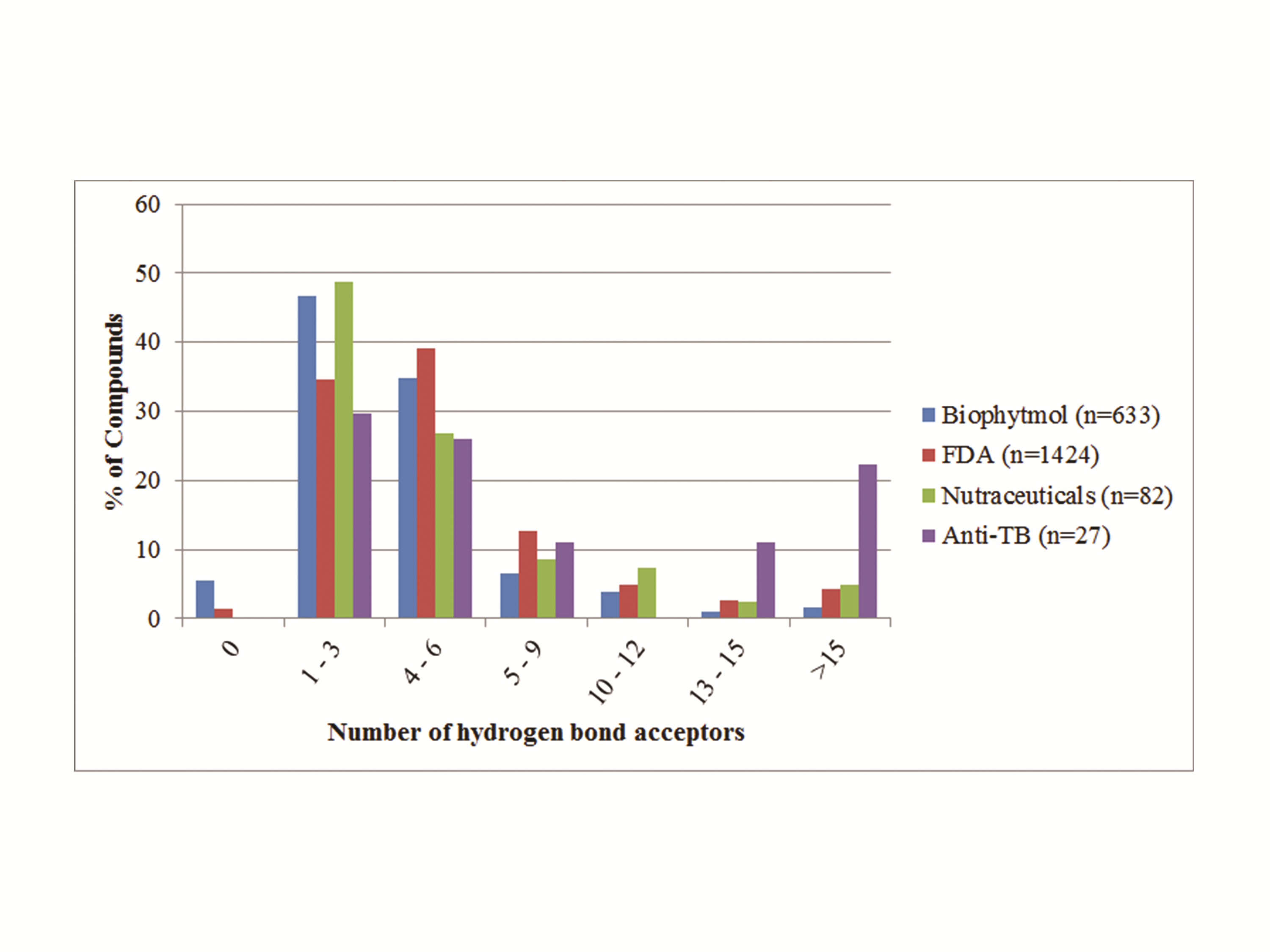

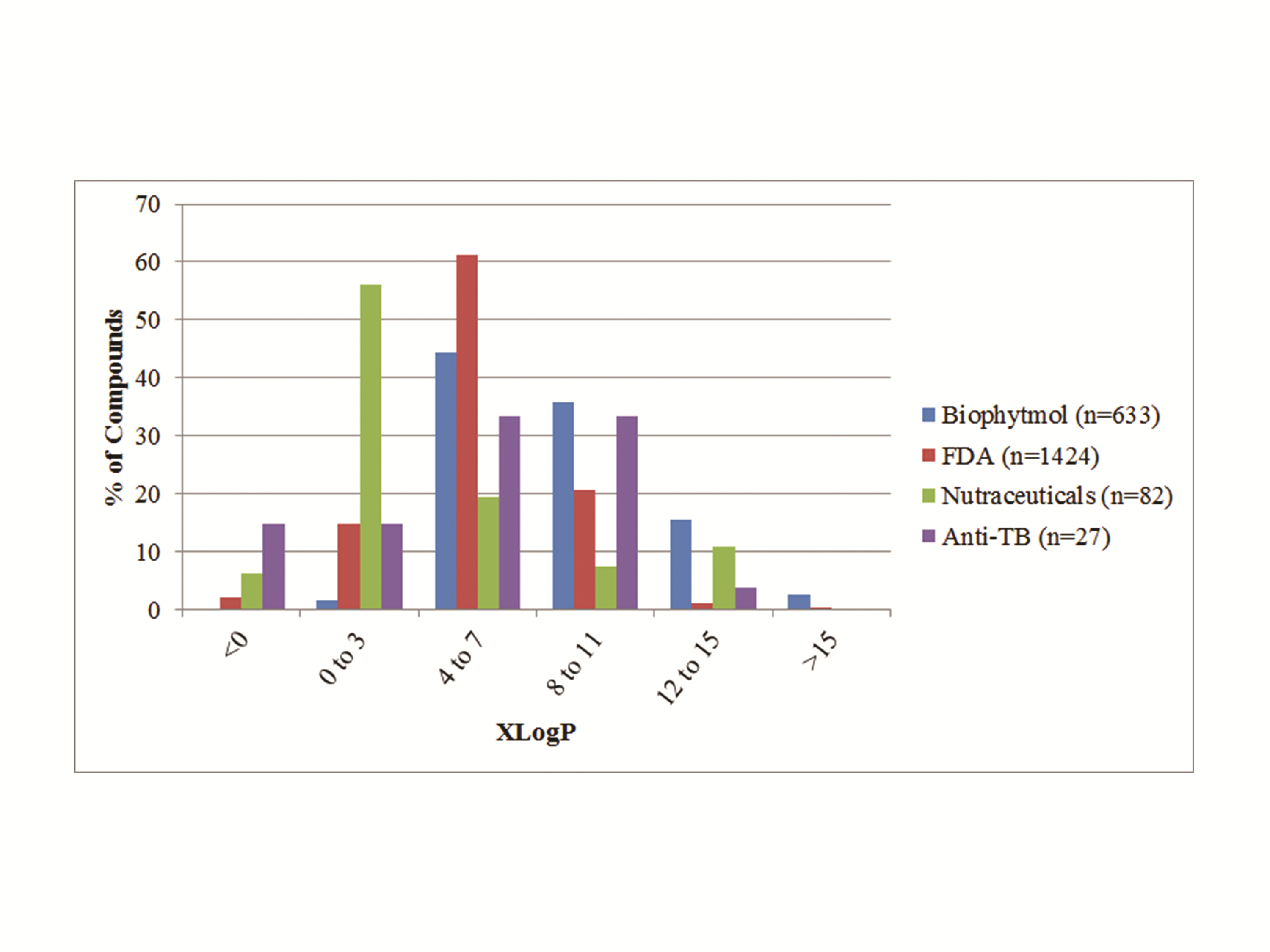

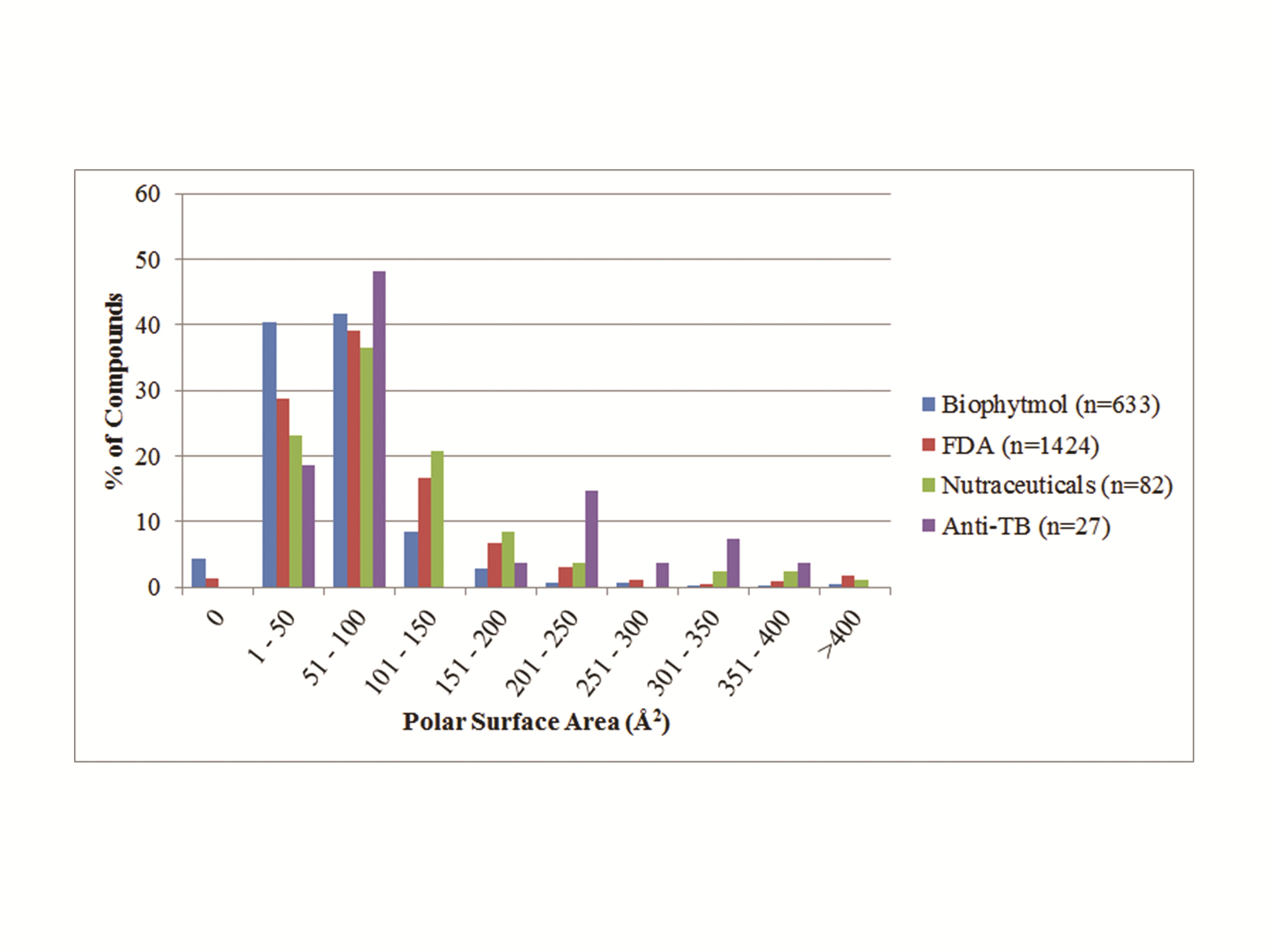

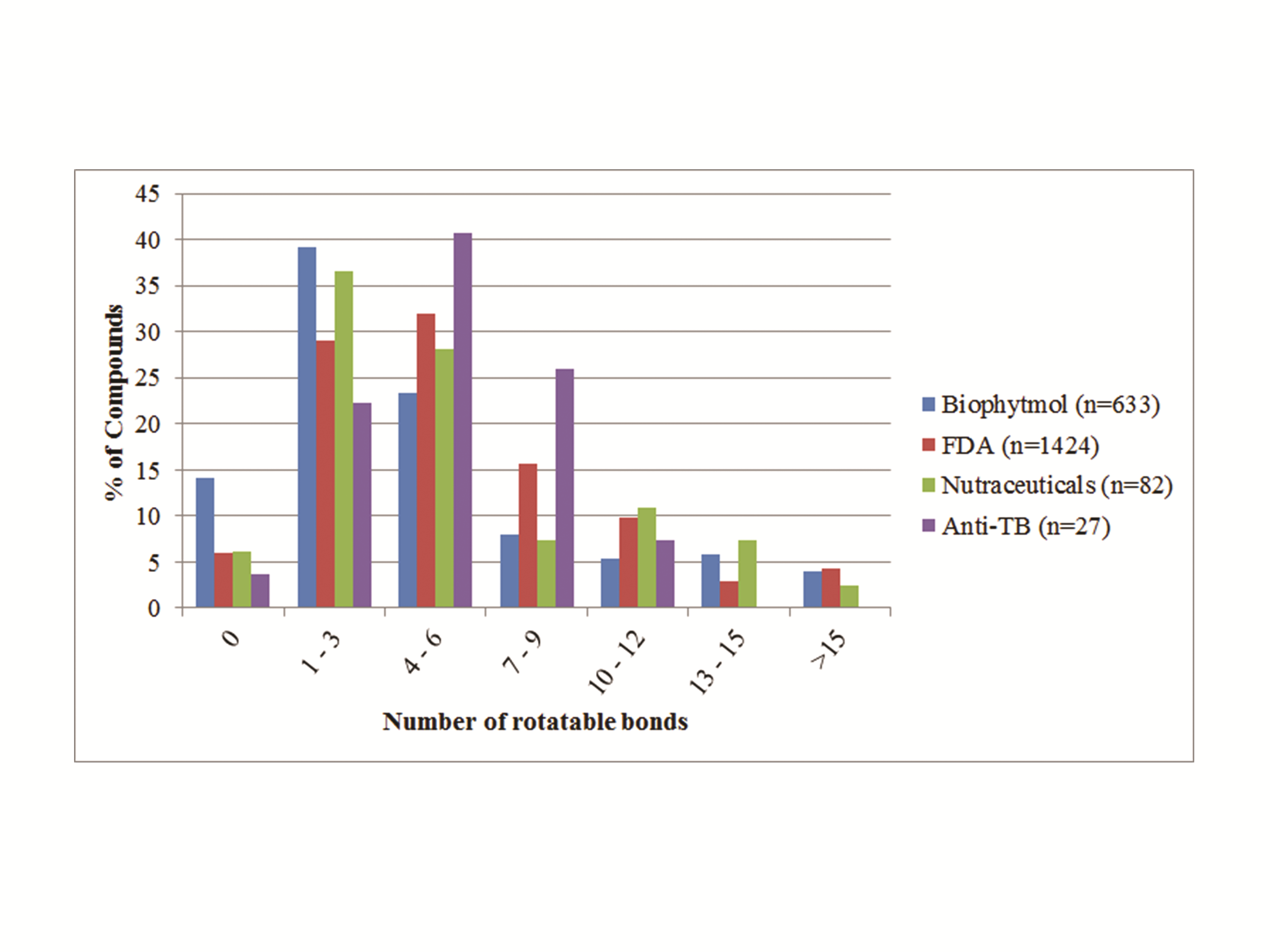
**

A

B

C

D

E

F

**Figure S6.** Distribution of physicochemical properties of BioPhytMol compounds with respect to different classes of drugs on the basis of **(A)** Molecular weight, **(B)** Hydrogen bond donors, **(C)** Hydrogen bond acceptors, **(D)** XLogP, **(E)** Polar Surface Area, **(F)** Number of rotatable bonds

**
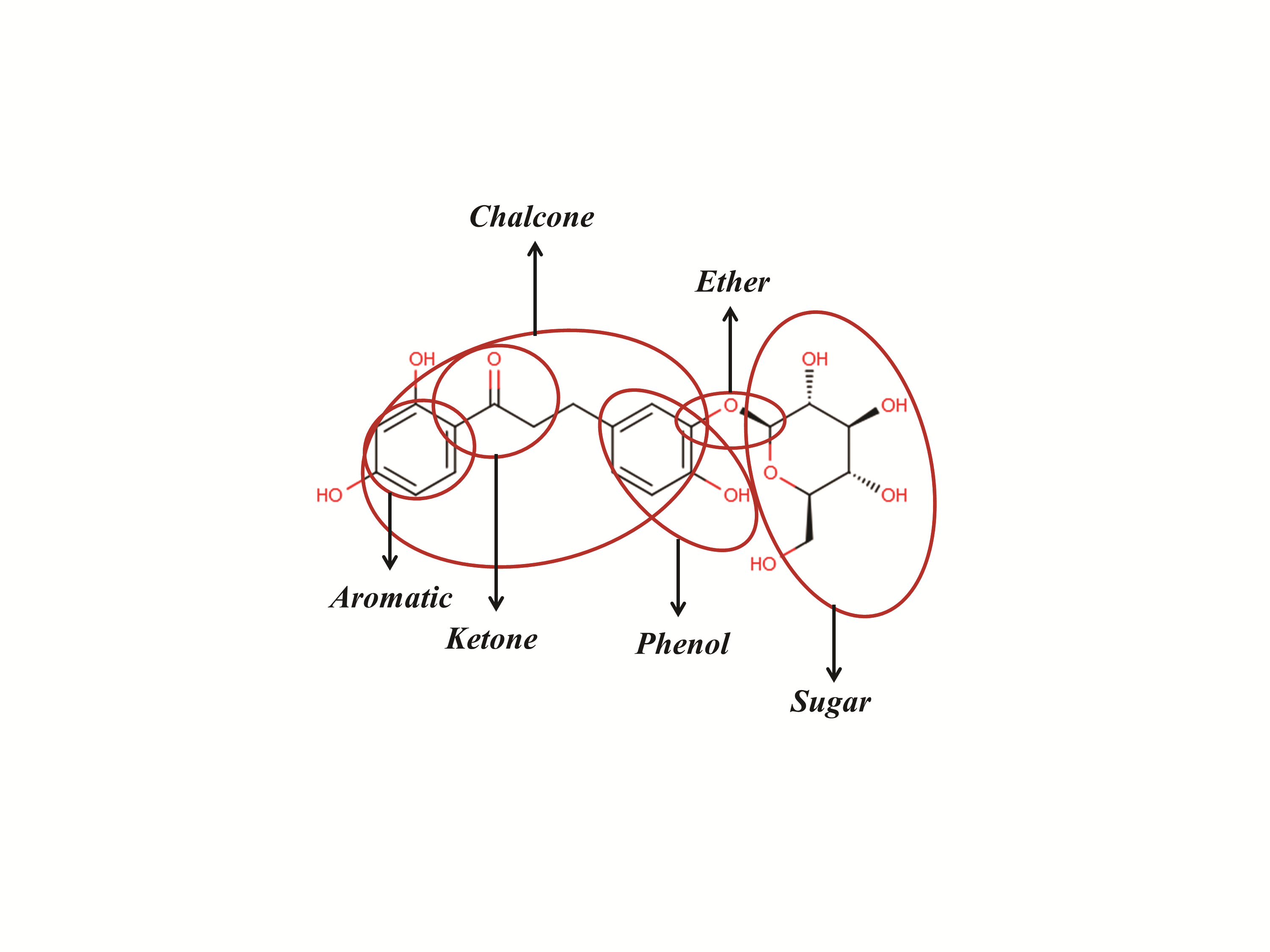
**

**Figure S7.** Proposed simplified ontology for chemical classification using ‘Dihydromonospermoside’ as an example.

**Table S1.** Existing anti-TB drugs classified according to their drug class and differentiated as per their origin (natural or synthetic). Eight drugs have microbial (natural product) origin while twenty have synthetic origin.

| **Line of treatment** | **Drugs** | **Natural** | **Synthetic** | **Reference** |
| --- | --- | --- | --- | --- |
| **First Line** | Isoniazid |  | ✓ |  |
|  | Rifampicin | ✓ |  |  |
|  | Pyrazinamide |  | ✓ |  |
|  | Ethambutol |  | ✓ |  |
|  | Rifapentine | ✓ |  |  |
| **Second Line** | Streptomycin | ✓ |  |  |
|  | Kanamycin | ✓ |  |  |
|  | Amikacin | ✓ |  |  |
|  | Capreomycin | ✓ |  |  |
|  | Viomycin | ✓ |  |  |
|  | Ciprofloxacin |  | ✓ |  |
|  | Levofloxacin |  | ✓ |  |
|  | Moxifloxacin |  | ✓ |  |
|  | Ofloxacin |  | ✓ |  |
|  | Gatifloxacin |  | ✓ |  |
|  | Para-aminosalicylic acid |  | ✓ |  |
|  | Cycloserine | ✓ |  |  |
|  | Terizidone |  | ✓ |  |
|  | Ethionamide |  | ✓ |  |
|  | Prothionamide |  | ✓ |  |
|  | Thiacetazone (synonym**: 4′-Formylacetanilide thiosemicarbazone)** |  | ✓ |  |
|  | Linezolid |  | ✓ |  |
| **Third Line** | Clofazimine |  | ✓ |  |
|  | Clarithromycin |  | ✓ |  |
|  | Amoxicillin plus Clavulanate |  | ✓ |  |
|  | Imipenem plus Cilastatin |  | ✓ |  |

**Table S2. Data structure and definition of data fields of BioPhytMol**

| **Field Name** | **Full Description of Field** |
| --- | --- |
| **Compound ID** | A unique identifier for a particular BioPhytMol record |
| **Compound Structure** | 2D and 3D structure of the phytomolecule in SDF and MOL format |
| **Plant Source and Common Name** | Information of the plant source from where the anti-mycobacterial phytomolecule/extract was derived and its corresponding common name |
| **Source Family** | Family of the plant source |
| **Origin** | Geographical location of the plant source |
| **Plant Part Used** | Part of the plant used for conducting experiments |
| **Extract** | Information on prepared extract |
| **Target Bacteria** | Bacterial Strains against which the activity of the phytomolecule/plant extract has been checked |
| **Assay/Test Done** | Name of the assay used to measure the activity of the active compound identified |
| **Positive Control Used** | Concentration of the reference controls used in the assay |
| **Inhibition [%]** | Percent inhibition of mycobacterial growth |
| **Activity [MIC]** | Minimum Inhibitory Concentration value (in µg/ml) |
| **Activity (In terms of dilution)** | Activity in terms of dilution |
| **Activity (Zone of inhibition in mm)** | Zone of inhibition (in mm) |
| **Active Compound Identified** | Name of the anti-mycobacterial phytomolecule identified from the plant source |
| **PubChem ID** | ID of the active compound identified present in PubChem database (may contain a different ID if not present in PubChem) |
| **Ethnomedicinal Information** | Medicinal value of the plant source |
| **PubMed ID** | ID of the reference paper of PubMed database, from where the data has been extracted |
| **Extract Preparation** | Steps followed for preparing the extract |
| **Chemical Classification** | Structural classification of the active compound identified |
| **Media/Broth Used** | Information on the type of medium used to culture mycobacteria |
| **Cytotoxicity Assay** | Information of assay used to test cytotoxicity |
| **Molecular Weight** | Calculated molecular weight of the active compound identified |
| **Molecular Formula** | Molecular formula of the active compound identified |
| **SMILES** | Representation of the active compound identified in SMILES format |
| **XLogP** | Calculated octanol/water partition coefficient of the active compound identified |
| **PSA** | Calculated polar surface area of the active compound identified |
| **H-bond Donor** | No. of electronegative atoms in the active compound identified |
| **H-bond Acceptor** | No. of electropositive atoms in the active compound identified |
| **No. of Rotatable Bond Count** | No. of single bonds between heavy atoms in the active compound identified |
| **No. of Rings** | No. of cycles in the active compound identified |
| **No. of N** | No. of Nitrogen atoms in the active compound identified |
| **No. of O** | No. of Oxygen atoms in the active compound identified |
| **No. of S** | No. of Sulphur atoms in the active compound identified |
| **Reference(s)** | Provides information of the reference sources from where the data has been collected |
| **Curator** | Provides Sysborg ID of the curator |

**Table S3:** BioPhytMol compounds (B_mols) showing similarity to various drug classes - anti-TB drugs (A_tb), FDA approved nutraceutical drugs (FDA_nutra) and FDA approved small molecule drugs (FDA_small) through ChemAxon’s near neighbours search method

| **Dissimilarity Threshold (T)** | **B_mols** | **A_tb** |
| --- | --- | --- |
| **T = 0.4** | 10 | 2 |
|  | **B_mols** | **FDA_nutra** |
| **T = 0.15** | 4 | 4 |
|  | **B_mols** | **FDA_small** |
| **T = 0.15** | 16 | 15 |

**Table S4.** Twenty B_mols near neighbours of two GSK compounds (GSK146660A and GSK1996236A) with known target information

| **S.No.** | **GSK Compound** | **Structure** | **Targets** | **Gene Description** | **BioPhytMol compound** | **Structure** | **Target bacteria** | **MIC (µg/ml)** |
| --- | --- | --- | --- | --- | --- | --- | --- | --- |
| 1 | GSK146660A | 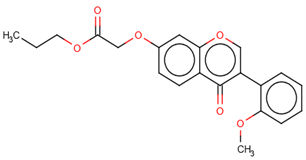 | Rv0458 | 'Probable aldehyde dehydrogenase (EC 1.2.1.3)' | Cajanin | 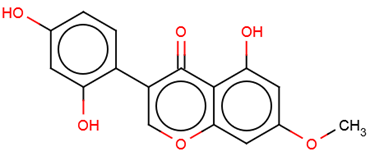 | *Mycobacterium tuberculosis* H37Rv | 110 |
|  |  |  | Rv3170 | 'Putative flavin-containing monoamine oxidase AofH (EC 1.4.3.-)' | Prunetin | 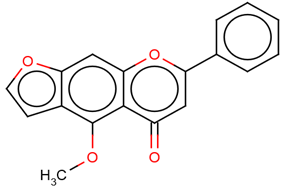 | *Mycobacterium tuberculosis* H37Rv | 30 |
|  |  |  |  |  | Genistein | 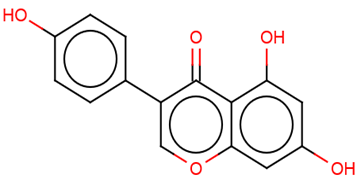 | *Mycobacterium tuberculosis* H37Rv | 35 |
|  |  |  |  |  | Formononetin | 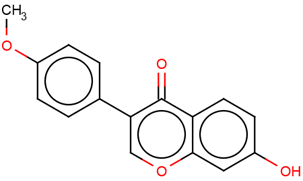 | *Mycobacterium tuberculosis* H37Rv | 50 |
|  |  |  |  |  | Afrormosin | 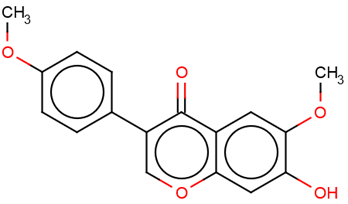 | *Mycobacterium tuberculosis* H37Rv | 25 |
|  |  |  |  |  | Dalparvone | 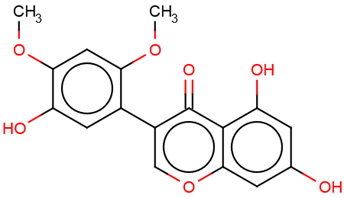 | *Mycobacterium tuberculosis* H37Rv | 50 |
| 2 | GSK1996236A | 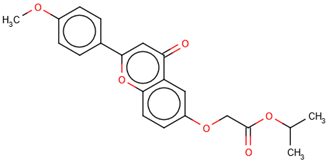 | Rv0458 | 'Probable aldehyde dehydrogenase (EC 1.2.1.3)' | Acacetin | 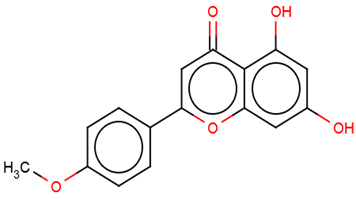 | *Mycobacterium tuberculosis* | 200 |
|  |  |  | Rv1747 | 'ABC transporter ATP-binding/permease protein Rv1747 (EC 3.6.3.-)' | Luteolin | 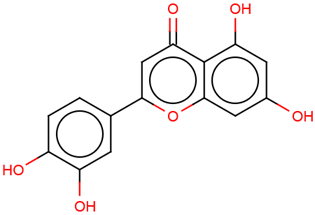 | *Mycobacterium tuberculosis* | 200 |
|  |  |  | Rv2971 | 'Uncharacterized oxidoreductase Rv2971/MT3049 (EC 1.-.-.-)' | Apigenin | 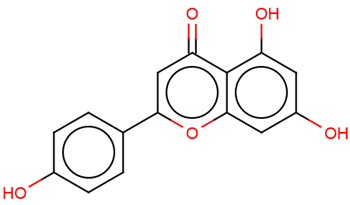 | *Mycobacterium tuberculosis* H37Rv | 70 |
|  |  |  | Rv3170 | 'Putative flavin-containing monoamine oxidase AofH (EC 1.4.3.-)' | 5, 7, 2` - Trihydroxyflavone | 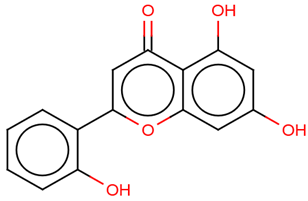 | *Mycobacterium tuberculosis* | 100 |
|  |  |  | Rv0548c | 4-Dihydroxy-2-naphthoyl-CoA synthase (DHNA-CoA synthase) (EC 4.1.3.36)' | 5 - Hydroxy - 3, 7, 4` - Trimethoxyflavone | 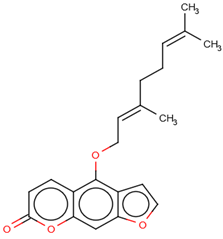 | *Mycobacterium tuberculosis* H37Rv | 100 |
|  |  |  |  |  | Kumatakenin | 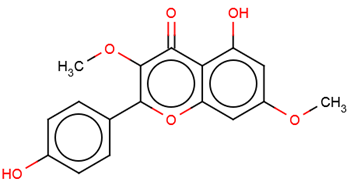 | *Mycobacterium tuberculosis* H37Rv | 100 |
|  |  |  |  |  | 3, 5, 7, 4' - Tetramethoxyflavone | 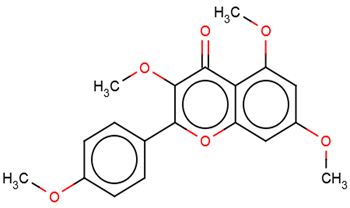 | *Mycobacterium tuberculosis* | 200 |
|  |  |  |  |  | 5, 7, 4` - Trimethoxyflavone | 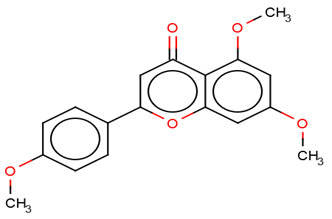 | *Mycobacterium tuberculosis* | 50 |
|  |  |  |  |  | Isothymusin | 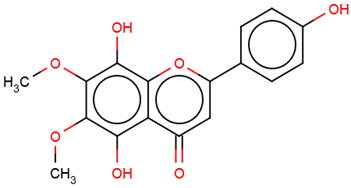 | *Mycobacterium tuberculosis* | 200 |
|  |  |  |  |  | Ermanin | 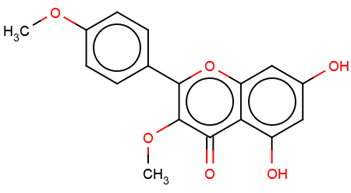 | *Mycobacterium tuberculosis* H37Rv (ATCC 27 294) | 100 |
|  |  |  |  |  | Pinnatin | 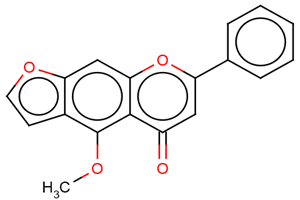 | *Mycobacterium tuberculosis* H37Ra | 12.5 |
|  |  |  |  |  | 7, 3' , 4' - Tri - Hydroxyflavone | 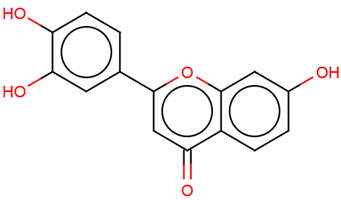 | *Mycobacterium tuberculosis* H37Ra | 50 |
|  |  |  |  |  | 3,7-Dimethoxyflavone | 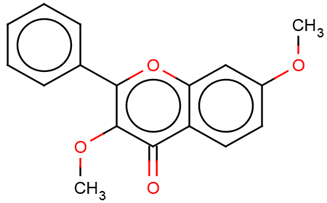 | *Mycobacterium tuberculosis* H37Ra | 50 |
|  |  |  |  |  | Nevadensin | 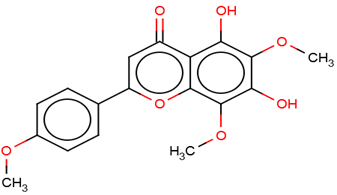 | *Mycobacterium tuberculosis* | 200 |

**Table S5.** BioPhytMol database fields mapped on the NCBO Natural products ontology (NATPRO)

| **BioPhytMol field** | **NATPRO Class** | **NATPRO Subclasses** | | | |
| --- | --- | --- | --- | --- | --- |
| **part_used** | [plant anatomical entity](http://bioportal.bioontology.org/ontologies/50642/?p=terms&conceptid=PO%3A0025131) | [plant structure](http://bioportal.bioontology.org/ontologies/50642/?p=terms&conceptid=PO%3A0009011) |  |  |  |
| **target_bacteria** | [biotopMaterialEntity](http://bioportal.bioontology.org/ontologies/46865/?p=terms&conceptid=biotopMaterialEntity) | [biotop:LivingOrganism](http://bioportal.bioontology.org/ontologies/46865/?p=terms&conceptid=biotop%3ALivingOrganism) | [biotop:OrganismByTaxonPartition](http://bioportal.bioontology.org/ontologies/46865/?p=terms&conceptid=biotop%3AOrganismByTaxonPartition) | [biotop:Prokaryote](http://bioportal.bioontology.org/ontologies/46865/?p=terms&conceptid=biotop%3AProkaryote) | [Bacteria](http://bioportal.bioontology.org/ontologies/46865/?p=terms&conceptid=Bacteria) |
| **activity** | [biotop:ImmaterialNonphysicalEntity](http://bioportal.bioontology.org/ontologies/46865/?p=terms&conceptid=biotop%3AImmaterialNonphysicalEntity) | [biotop:Measure](http://bioportal.bioontology.org/ontologies/46865/?p=terms&conceptid=biotop%3AMeasure) | [ActivityMeasure](http://bioportal.bioontology.org/ontologies/46865/?p=terms&conceptid=ActivityMeasure) |  |  |
| **activity_in_dilutn** | [biotop:ImmaterialNonphysicalEntity](http://bioportal.bioontology.org/ontologies/46865/?p=terms&conceptid=biotop%3AImmaterialNonphysicalEntity) | [biotop:Measure](http://bioportal.bioontology.org/ontologies/46865/?p=terms&conceptid=biotop%3AMeasure) | [ActivityMeasure](http://bioportal.bioontology.org/ontologies/46865/?p=terms&conceptid=ActivityMeasure) |  |  |
| **inhibition_zone** | [biotop:ImmaterialNonphysicalEntity](http://bioportal.bioontology.org/ontologies/46865/?p=terms&conceptid=biotop%3AImmaterialNonphysicalEntity) | [biotop:Measure](http://bioportal.bioontology.org/ontologies/46865/?p=terms&conceptid=biotop%3AMeasure) | [ActivityMeasure](http://bioportal.bioontology.org/ontologies/46865/?p=terms&conceptid=ActivityMeasure) |  |  |
| **ethno_med_info** | [biotop:condition](http://bioportal.bioontology.org/ontologies/46865/?p=terms&conceptid=biotop%3Acondition) | [biotop:PathologicalCondition](http://bioportal.bioontology.org/ontologies/46865/?p=terms&conceptid=biotop%3APathologicalCondition) | [disease by infectious agent](http://bioportal.bioontology.org/ontologies/46865/?p=terms&conceptid=DOID_0050117) | [bacterial infectious disease](http://bioportal.bioontology.org/ontologies/46865/?p=terms&conceptid=DOID_104) |  |
| **pubmed_source_lit** | [biotop:ImmaterialNonphysicalEntity](http://bioportal.bioontology.org/ontologies/46865/?p=terms&conceptid=biotop%3AImmaterialNonphysicalEntity) | [biotop:IntellectualProduct](http://bioportal.bioontology.org/ontologies/46865/?p=terms&conceptid=biotop%3AIntellectualProduct) | [RepositorySource](http://bioportal.bioontology.org/ontologies/46865/?p=terms&conceptid=RepositorySource) |  |  |
| **chem_class** | [biotopMaterialEntity](http://bioportal.bioontology.org/ontologies/46865/?p=terms&conceptid=biotopMaterialEntity) | [biotop:OrganicMolecularEntityPartition](http://bioportal.bioontology.org/ontologies/46865/?p=terms&conceptid=biotop%3AOrganicMolecularEntityPartition) |  |  |  |
| **tar_pro** | [biotop:Role](http://bioportal.bioontology.org/ontologies/46865/?p=terms&conceptid=biotop%3ARole) | [biotop:ChemicalRole](http://bioportal.bioontology.org/ontologies/46865/?p=terms&conceptid=biotop%3AChemicalRole) | [Target](http://bioportal.bioontology.org/ontologies/46865/?p=terms&conceptid=Target) |  |  |
| **conc_pos_control** | [biotop:ImmaterialNonphysicalEntity](http://bioportal.bioontology.org/ontologies/46865/?p=terms&conceptid=biotop%3AImmaterialNonphysicalEntity) | [biotop:Measure](http://bioportal.bioontology.org/ontologies/46865/?p=terms&conceptid=biotop%3AMeasure) | [ActivityMeasure](http://bioportal.bioontology.org/ontologies/46865/?p=terms&conceptid=ActivityMeasure) |  |  |
| **cyto_assay** | [biotopMaterialEntity](http://bioportal.bioontology.org/ontologies/46865/?p=terms&conceptid=biotopMaterialEntity) | [biotop:Cell](http://bioportal.bioontology.org/ontologies/46865/?p=terms&conceptid=biotop%3ACell) | [biotop:CellByLocusPartition](http://bioportal.bioontology.org/ontologies/46865/?p=terms&conceptid=biotop%3ACellByLocusPartition) | [biotop:ModifiedCell](http://bioportal.bioontology.org/ontologies/46865/?p=terms&conceptid=biotop%3AModifiedCell) | [CellLine](http://bioportal.bioontology.org/ontologies/46865/?p=terms&conceptid=CellLine) |
| **ref1, ref2, ref3** | [biotop:ImmaterialNonphysicalEntity](http://bioportal.bioontology.org/ontologies/46865/?p=terms&conceptid=biotop%3AImmaterialNonphysicalEntity) | [biotop:IntellectualProduct](http://bioportal.bioontology.org/ontologies/46865/?p=terms&conceptid=biotop%3AIntellectualProduct) | [RepositorySource](http://bioportal.bioontology.org/ontologies/46865/?p=terms&conceptid=RepositorySource) |  |  |

References

1. Sycheva TP, Pavlova TN, Shchukina MN: **Synthesis of isoniazid from 4-cyanopyridine.** *Pharm Chem J* 1972, **6:**696-698.

2. Sensi P, Margalith P, Timbal MT: **Rifomycin, a new antibiotic; preliminary report.** *Il Farmaco; edizione scientifica* 1959, **14:**146-147.

3. Kushner S, Dalalian H, Sanjurjo JL, Bach FL, Safir SR, Smith VK, Williams JH: **Experimental Chemotherapy of Tuberculosis. II. The Synthesis of Pyrazinamides and Related Compounds1.** *Journal of the American Chemical Society* 1952, **74:**3617-3621.

4. Singh B: Synthesis of ethambutol. Google Patents; 1976.

5. Grayson ML, Crowe SM, McCarthy JS, Mills J, Mouton JW, Norrby SR, Paterson DL, Pfaller MA: *Kucers' The Use of Antibiotics Sixth Edition: A Clinical Review of Antibacterial, Antifungal and Antiviral Drugs.* Taylor & Francis; 2010.

6. Bruton J, Horner WH: **Biosynthesis of Streptomycin: III. ORIGIN OF THE CARBON ATOMS OF STREPTOSE.** *Journal of Biological Chemistry* 1966, **241:**3142-3146.

7. Garrod LP, O'Grady F, Lambert HP: *Antibiotic and chemotherapy.* Churchill Livingstone, Edinburgh, New York; 1981.

8. Stevens P, Young LS, Hewitt WL: **125I-Radioimmunoassay of amikacin and comparison with a microbioassay.** *The Journal of antibiotics* 1976, **29:**829-832.

9. Herr JEB, Hamill RL, Mcguire JM: Capreomycin and its preparation. Google Patents; 1964.

10. Barkei JJ, Kevany BM, Felnagle EA, Thomas MG: **Investigations into viomycin biosynthesis by using heterologous production in Streptomyces lividans.** *Chembiochem : a European journal of chemical biology* 2009, **10:**366-376.

11. Grohe K, Zeiler HJ, Metzger KG: 1-cyclopropyl-6-fluoro-1,4-dihydro-4-oxo-7-piperazino- quinoline-3-carboxylic acids, a process for their preparation and antibacterial agents containing these compounds. Google Patents; 1987.

12. Yang Z, Ye W, Zhang W: Methods for preparation of levofloxacin and ofloxacin. Google Patents; 2005.

13. Bosché P, Mahler HF, Weisemann C: Pharmaceutical moxifloxacin preparation. Google Patents; 2003.

14. Niddam-Hildesheim V, Dolitzky BZ, Pilarski G, Sterimbaum G: Synthesis of gatifloxacin. Google Patents; 2009.

15. Centolella AP: p-aminosalicylic acid. Google Patents; 1958.

16. Li X, Meng X, Duan H, Wang L, Wang S, Zhang Y, Qin D: **Original and efficient synthesis of D-cycloserine.** *Archiv der Pharmazie* 2010, **343:**473-475.

17. Vora A: **Terizidone.** *The Journal of the Association of Physicians of India* 2010, **58:**267-268.

18. Yakhontov LN, Azimov VA, Sycheva TP, Aryuzina VM, Sakovich TV, Shchukina MN: **A new method of synthesizing the antitubercular drug prothionamide.** *Pharm Chem J* 1976, **10:**227-230.

19. Jack B, Lott WA, Wiselogle FY: p-isoamoxy-benzaldehyde thiosemicarbazone. Google Patents; 1955.

20. Barbachyn MR, Brickner SJ, Hutchinson DK: Substituted oxazine and thiazine oxazolidinone antimicrobials. Google Patents; 1997.

21. Vardanyan R, Hruby V: *Synthesis of Essential Drugs.* Elsevier Science; 2006.

22. Kansal VK, Mistry DN, Gandhi M, Patel RR: Process for the preparation of clarithromycin. Google Patents; 2009.

23. Grossman JH, Hardcastle GA: Production of amoxicillin. Google Patents; 1976.

24. Cole M, Howarth TT, Reading C: Process for the production of clavulanic acid. Google Patents; 1978.

25. Kumar Y, Tewari N, Rai BP: Process for the preparation of imipenem. Google Patents; 2008.

26. Kumar Y, Pandey A, Srivastava TK, Tyagi OD: Process for the preparation of cilastatin. Google Patents; 2003.
