# Supplementary figures and images for: BioPhytMol: a drug discovery community resource on anti-mycobacterial phytomolecules and plant extracts
Source: J Cheminform. 2014 Oct 11;6:46. doi: 10.1186/s13321-014-0046-2 (PMC4206768; doi:10.1186/s13321-014-0046-2)

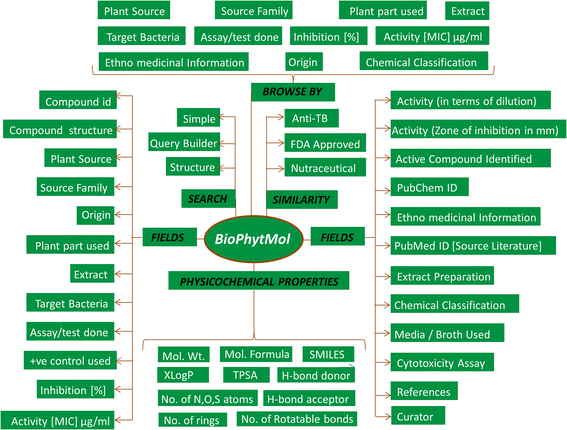

Supplement: Supplementary file 2 — Authors’ original file for figure 1 [file 13321_2014_46_MOESM2_ESM.gif]

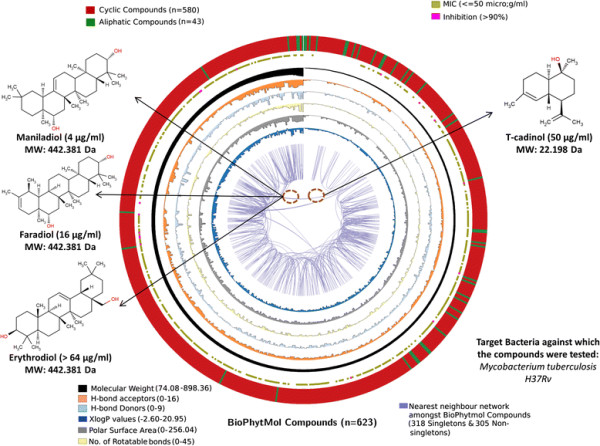

Supplement: Supplementary file 3 — Authors’ original file for figure 2 [file 13321_2014_46_MOESM3_ESM.jpeg]

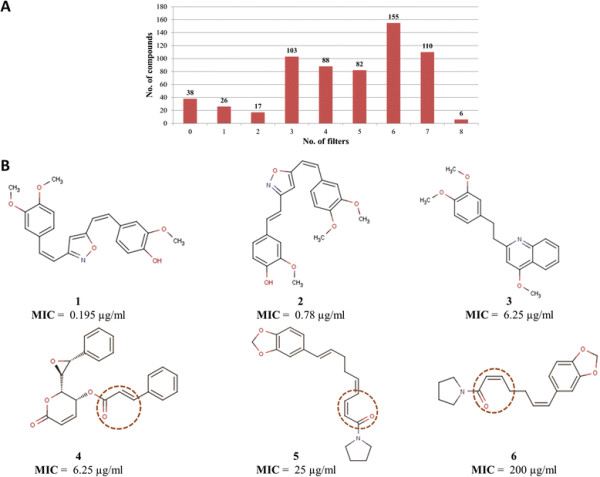

Supplement: Supplementary file 4 — Authors’ original file for figure 3 [file 13321_2014_46_MOESM4_ESM.jpeg]

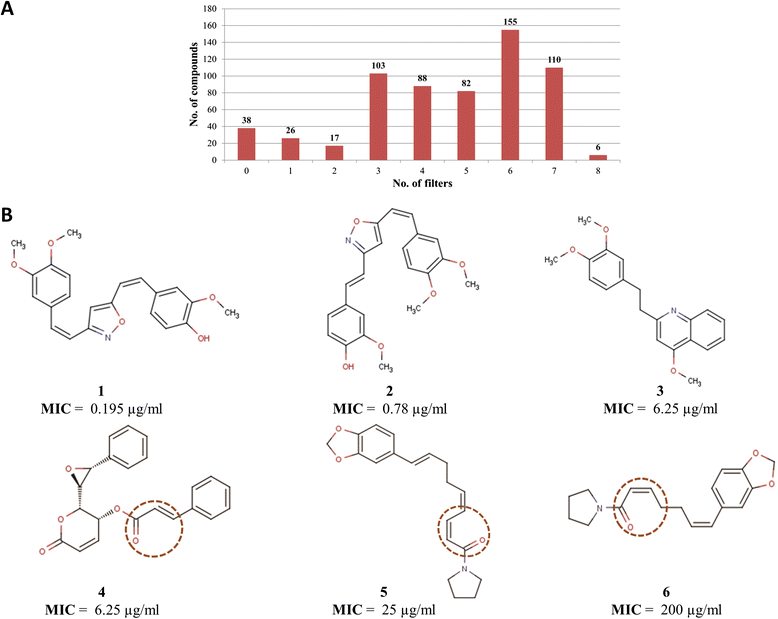

Supplement: Supplementary file 5 — Authors’ original file for figure 4 [file 13321_2014_46_MOESM5_ESM.gif]
